# Time-Lapse In Situ 3D Imaging Analysis of Human Enamel Demineralisation Using X-ray Synchrotron Tomography

**DOI:** 10.3390/dj11050130

**Published:** 2023-05-09

**Authors:** Cyril Besnard, Ali Marie, Sisini Sasidharan, Robert A. Harper, Shashidhara Marathe, Jonathan Moffat, Richard M. Shelton, Gabriel Landini, Alexander M. Korsunsky

**Affiliations:** 1 MBLEM, Department of Engineering Science, University of Oxford, Parks Road, Oxford OX1 3PJ, UKalexander.korsunsky@eng.ox.ac.uk (A.M.K.); 2 School of Dentistry, University of Birmingham, 5 Mill Pool Way, Edgbaston, Birmingham B5 7EG, UK; 3 Diamond Light Source Ltd., Didcot OX11 0DE, UK; 4 Oxford Instruments Asylum Research, High Wycombe HP12 3SE, UK

**Keywords:** human enamel, in situ demineralisation, synchrotron X-ray tomography, image analysis, AFM

## Abstract

Caries is a chronic disease that causes the alteration of the structure of dental tissues by acid dissolution (in enamel, dentine and cementum) and proteolytic degradation (dentine and cementum) and generates an important cost of care. There is a need to visualise and characterise the acid dissolution process on enamel due to its hierarchical structure leading to complex structural modifications. The process starts at the enamel surface and progresses into depth, which necessitates the study of the internal enamel structure. Artificial demineralisation is usually employed to simulate the process experimentally. In the present study, the demineralisation of human enamel was studied using surface analysis carried out with atomic force microscopy as well as 3D internal analysis using synchrotron X-ray tomography during acid exposure with repeated scans to generate a time-lapse visualisation sequence. Two-dimensional analysis from projections and virtual slices and 3D analysis of the enamel mass provided details of tissue changes at the level of the rods and inter-rod substance. In addition to the visualisation of structural modifications, the rate of dissolution was determined, which demonstrated the feasibility and usefulness of these techniques. The temporal analysis of enamel demineralisation is not limited to dissolution and can be applied to other experimental conditions for the analysis of treated enamel or remineralisation.

## 1. Introduction

Caries are a worldwide problem that causes damage to dental tissue, affecting billions of people [1,2]. Numerous studies have been dedicated to the investigation of enamel caries, but there remains a lack of therapeutic methods to reverse the damage. The comprehensive characterisation of structural changes in enamel from the micro to nanoscale during disease progression is essential to support the development of new treatments. Many novel analytical techniques have been able to push the limits of resolution in this field.

The dissolution of enamel in caries results from the effect of acid produced by bacteria (e.g., *Streptococcus mutans* (*S. mutans*)) [3,4,5] and the inhomogeneous structure of the enamel [6,7]. To be able to study this slow anisotropic dissolution process, artificial etching (using acidic solutions) has been widely used to simulate this dynamic process. Recently, analysis using synchrotron radiation, particularly X-ray micro-computed tomography, has been used to examine the natural and artificial demineralisation of human enamel with high-resolution 3D imaging. A detailed statistical analysis of the structure of normal and carious enamel has also been reported recently, including the localised visualisation and quantification of the demineralised regions [6,8]. Furthermore, the fast acquisition (less than 30 min) for large datasets using X-ray micro-computed tomography has revealed a need to understand structural modifications at all scale levels [9]. This study expands on our previous findings, which analysed enamel in 3D [6,8], and extends the scope by performing in situ analysis using synchrotron X-ray tomography in a microfluidic setup to provide methods for characterising the dissolution of enamel. 

Enamel has a hierarchical structure [6,8,10,11,12], which requires a multi-scale study for its full characterisation. It is mainly constituted of minerals and a small percentage of water and protein [13,14]. At the nanoscale, it is composed of mineral hydroxyapatite nanocrystallites [15,16] with different orientations [17,18]. At the micro-scale, these crystallites are organised into rods and inter-rod substances with dimensions of around 5 and 2 µm, respectively, which are organised in an ordered, although not a perfectly repetitive pattern [10,15,19,20,21]. At larger scales, enamel features change in the mineralisation density known as the striae of Retzius and also undergo variations due to the changes in the direction of the rods, known as the Hunter–Schreger bands (HSBs) [22,23]. Enamel has outstanding mechanical and thermal properties; however, its resistance to acids is very low, which results in the removal of ions (calcium and phosphate), the formation of pores, and, eventually, cavitation. While research has been conducted to understand the origin of demineralisation at the nanoscale level [16,18,24], the visualisation and analysis of the progression of demineralisation in 3D at a high-resolution and high speed have not yet been extensively detailed to date in dental tissues [25,26].

Enamel caries is a dynamic process of the dissolution and precipitation of minerals, conditioned by the environmental pH [27], that requires not only post-dissolution analysis but also in situ characterisation to characterise the pathway of the acid and the progression of enamel dissolution. In a static condition, the structure has been analysed using microscopy techniques, including scanning electron microscopy (SEM) [28], focused ion beam (FIB)-SEM [6], atomic force microscopy (AFM) [29], and transmission electron microscopy to study preferential demineralisation in nanocrystallites [30]. X-rays were also applied to study the enamel, including radiography [31], tomography [8], ptychography and XRF, covering structural and chemical details [18]. 

Dynamic experiments were implemented in the study of enamel and caries. While AFM provided details on the onset of dissolution [32,33,34,35,36], as well as the metabolic activities of *S. mutans* [37], there was a lack of information regarding the dissolution volume in large regions. Radiography with fast acquisitions has been used to study the time-lapse evolution of greyscale data from enamel using fast scans; however, the time-lapse of 3D structural details was not apparent [31]. 

In addition to imaging, in situ wide- and small-angle X-ray scattering (WAXS and SAXS) analyses were used to determine the orientation and dimension of crystals during dissolution but did not allow visualisation [38]. In situ X-ray tomography has been used for various applications and provides abundant information on the modifications of structures in 3D. To analyse the demineralisation process over time, there is a requirement to develop and set up the use of fluidic devices that could lead to the development of four-dimensional (4D) tomography and provide structural information across time points [39,40].

Previously, in situ tomography was used to examine various materials such as the structure in bones [41], the process of alloy solidification [42,43,44], ice cream and magma crystallisation [45,46], solid electrolyte [47], magma [48], as well as corrosion [49]. The evolution of the dissolution of enamel has been studied using standard X-ray micro-computed tomography and for remineralisation in fluoride after one week [50]. The demineralisation of enamel was studied with 2.6 h per tomogram and a voxel size of 30 µm [51] correlated with the enamel structure at scales of around 4–5 rods/inter-rods. In vitro experiments were also conducted with teeth exposed to acid at different times [52]. However, for real-time 3D analysis, conventional X-ray imaging was not suitable as it could not analyse dynamic changes with a high-resolution and rapid acquisition. Recently, this combination of fast acquisition per tomogram and high-resolution using synchrotron beams was used to study the demineralisation and dimensions of dentine tubules with time [26].

In the present study, in situ analysis using synchrotron X-ray tomography in combination with fluidics to analyse the progression of enamel dissolution was performed. This process was also examined using in situ AFM analysis. Physical information, such as the rate of dissolution, area and volume, was extracted from tomography data to examine gradual structural changes in local regions of enamel. To the best of our knowledge, this is the first report covering various visualisations and analyses on human enamel examined in situ with tomography at a high resolution with less than a 30 min gap between datasets, with a voxel size of 325 nm and a field of view of 0.83 × 0.7 mm. This innovative method to study enamel can also be applied to other applications, such as enamel remineralisation and metal corrosion.

## 2. Methods

### 2.1. SEM

A sample containing enamel and dentine was analysed using an SEM Tescan Lyra 3 (Tescan, Czech Republic) with a voltage of 5 keV. The sample was polished down to 0.1 µm using diamond suspension and then mounted on an SEM stub for imaging.

### 2.2. AFM

Atomic force microscopy topography measurements were acquired on a Cypher ES from Oxford Instruments Asylum Research (OIAR) with an FS-1500 probe that was also from OIAR. The microscope was equipped with a liquid perfusion cell that allowed the exchange of buffers during measurements. A sample was measured in different pH solutions (2.2–10% *v*/*v* lactic acid; 4.3 acetate buffer—2.2 mM calcium, 2.2 mM phosphate, 75 mM acetate) [31,53,54] and in dry conditions at room temperature. For time-lapse measurements, images were acquired at a rate of 36 s per frame. The remaining images were acquired at a rate of 52 and 128 s per frame. Scans from 30.1 × 30.1 µm to ~1.7 × 1.7 µm were acquired at a resolution of 256 × 256 pixels. AFM topography images and corresponding height profiles from line sections were studied, with a step of 64 nm as well as the details of root mean square roughness Rq and average roughness Ra. The images were analysed with Gwyddion, OIAR software, Matlab and OriginPro.

### 2.3. Experimental Setup

#### 2.3.1. Sample Preparation for Tomography Analysis, Optical Profilometry and AFM 

Pristine human third molars extracted for non-caries were related to therapeutic reasons (National Research Ethics Committee; NHS-REC ref. 14/EM/1128, ref BCHCDent 332.1531.TB). The samples were fixed in a buffered solution of 10% formalin (Sigma Aldrich, Merk, Dorset, UK) after the removal of the root tips using a low-speed rotating diamond saw (IsoMet, Buehler, Germany). For synchrotron analysis, the tooth was removed from the sterilizing solution after four days and rinsed under running water before being sectioned radially into a thick slice with an intact outer enamel surface (an IsoMet diamond wafering blade with the bone saw to cut the tooth section). A flat-tipped 300 μm diameter needle (Septodont, Saint-Maur-des-Fossés, France) was clamped perpendicularly on the intact outer enamel surface, and the remaining surfaces were coated with a commercially available nail varnish mixed with methylene blue for better visualisation as shown in Supplementary Materials Figure S1a. An exposed window of a ~300 μm diameter circular non-varnished area was observed on the slice upon the removal of the needle (Supplementary Materials Figure S1a). The samples were stored in phosphate-buffered saline (PBS) at room temperature before further use. 

Prior to the synchrotron experiment, the sample was mounted on a plastic stick fixed inside an in-house custom-designed process cell (Supplementary Materials Figure S1b). The process cell was a 2 mL polypropylene test tube with an inlet and an outlet. The outlet was linked to a waste liquid collection container via long flexible tubing with a micro-valve. The upper inlet was connected to a luer fitting onto which a normal syringe was used to add the experimental liquid in the static mode at room temperature. The process cell was filled with PBS prior to use. An acidic solution of lactic acid served as the test liquid for simulating the cariogenic environment with a reasonable timescale in line with the synchrotron experiments and lactic acid solution (10% volume) at pH 2.2 [31]. The sample before the experiment was imaged using an optical profilometer, Alicona profilometer (Bruker, Coventry, UK).

For AFM, a tooth slice was cut (Diamond wafering blade, Buehler, Leinfelden-Echterdingen, Germany, IsoMet, Buehler, Leinfelden-Echterdingen, Germany) and polished down to 0.1 µm (Struers, Cleveland, OH, USA, Spectrographic Limited, Guiseley, UK) prior to analysis.

#### 2.3.2. Synchrotron Beamline Experiment

X-ray micro-tomography experiments were performed at the Diamond Manchester Imaging Branchline (I13-2, Diamond Light Source (Harwell, Didcot, UK)) [55,56]. The experiment was carried out using a modified method based on our previous work on static conditions at this beamline [6,8,12]. A lactic acid solution at pH 2.2 was injected into the flow cell, where the sample was exposed to acid for 10 h in a closed container. Several consecutive X-ray synchrotron tomography acquisitions were carried out at an interval of ~25 min (time of one acquisition), from 0 to 180° for 2500 projections with an acquisition time of 500 ms using ‘pink X-ray beam’ (weighted mean energy of 22 keV with a standard deviation of 3.5 keV). For each tomogram, the sample container was set up and rotated continuously in synchronisation with the camera data acquisition (called flyscan mode) to obtain the set of projections for each tomogram. Before the projections were collected, dark field and flat field images were acquired. A 10x objective lens pco.edge camera was used with a maximum field of view of 0.83 × 0.7 mm to obtain high-resolution scans with a voxel size of 0.325 μm. The study was carried out at room temperature without flow to study the feasibility of the analysis. However, to extend the analysis of caries, more research is needed at 37 °C, with the addition of flow and other solutions (e.g., artificial saliva [57]) to approach the condition of the oral environment.

#### 2.3.3. Analysis of the Tomography Data

Reconstructions were made using Savu and plugins, and the process list was similar to our previous work [8]. An analysis of the reconstructed data and projections was carried out using Avizo software (Thermo Fisher Scientific, Waltham, MA, USA) and imageJ/Fiji [58,59,60]. Prior to the analysis of the 3D data at different points, a manual alignment of the dataset was carried out at each time point, followed by a rigid 3D alignment in Avizo. Finally, the dataset coordinate was transformed to match the reference volume, which was the first time point of the data analysed. This led to a dataset series with the same dimensions and coordinates, including 1280 × 871 × 550 pixels (415.675 × 282.75 × 178.425 µm). The volumetric data were reduced and filtered using a median and a non-local mean filter to decrease the noise. A region of interest was then defined, 812 × 514 × 410 pixels (263.575 × 166.725 × 132.925 µm), and then the caries lesion was segmented (Supplementary Materials Figure S2). Additional details of the process for the reconstruction and analysis can be found in [6,8], with details on the thickness, Euclidean distance in 3D, and other 2D and 3D analyses of tomography data. 

Using the experiment within an in situ/operando setup, each tomogram acquired was defined as a time point, which created a time-lapse of the process that was rich in information. Data in each slice, volume and time could be analysed (as detailed in this section). The time reference was defined as $t_{0}$, and $t_{i}$ or $t_{i-1}$ for the other time points. Two types of measurement were obtained, including incremental (difference from two successive data points or non-consecutive data points, for instance) and absolute, from the reference dataset with each time point data. As an initial reference, the first dataset corresponded at time $t_{0}$ = 122 min. This time corresponded with the exposure of acid and the record time of the final projections per tomogram.

From the segmented region of the demineralisation region, several measurements were extracted ‘radius distance’ on extracted slices, and the area and volume were studied.

- ‘Radius distance’ is the distance from one point (equivalent to a seed) on the virtual slice to the last segmented data on the same slice at a specific angle. This was computed using Matlab and is detailed in Supplementary Materials Figure S3. This analysis can be conducted either on the reconstructed virtual slice after segmentation or on the projection after segmentation. The total distance at each time point could be determined, and then two other pieces of information on the distances were obtained: the absolute distance dabs at a certain time $t_{i}$ from a reference dataset, here $t_{0}$, and the cumulative distance dcum:
  $$dabs=d_{t_{i}\;}-d_{t_{0}\;}\;\text{µ}m\;\;dcum=d_{t_{i}\;}-d_{t_{\left(i-1\right)}\;}\;\text{µ}m$$
  Statistics data can be obtained if several points are taken into consideration.
  Distance difference at each time point analysed dacc%:
  $$dacc\%=\frac{d_{t_{i}}-d_{t_{0}}}{d_{t_{i}}}\%$$

From these values of distances and the temporal resolution, two rates of progression of demineralisation along these lines could be calculated:

$$dabsr=\frac{d_{t_{i}}-d_{t_{0}}}{t_{i}-t_{0}}\;\text{µ}m\cdot s^{-1}\;dcumr=\frac{d_{t_{i}}-d_{t_{\left(i-1\right)}\;}}{t_{i}-t_{\left(i-1\right)}}\;\text{µ}m\cdot s^{-1}$$

- The area with the temporal evolution of the surface of the lesion in virtual slices could be identified from 2D information. The total area per plane of the demineralised region referred to as $Area_{t_{i}}$ in µm^2^ was extracted, as well as the ratio of the area at time $t_{i},$ which was referred to as $Area_{t_{i}}$ and the initial area demineralised at $t_{0}$, $Area_{t_{0}}$, and was annotated Areafd:
  $$Areafd=\frac{Area_{t_{i}}}{Area_{t_{0}}}$$

The normalisation of the data was carried out with the percentage of increase in the demineralised area with time from the reference time $t_{0}$, Area_abs%:

$$Area\_abs\%=\frac{Area_{t_{i}}-Area_{t_{0}}}{Area_{t_{0}}}\times 100\;\mathrm{in}\;\%$$

and the percentage of increase between each time from the cumulative measurements between two increments, Area%:

$$Area\%=\frac{Area_{t_{i}}-Area_{t_{i-1}}}{Area_{t_{i}}}\times 100\mathrm{in}\%$$

In addition to the details of the area of the demineralised region at a different time point, the progression was evaluated based on the total area of the virtual slice used $Area_{tote}$ (equivalent to the non-demineralised region, total enamel region). Each virtual slice analysed contained an enamel region and different degrees of demineralisation, and this fraction was compared with the time and was referred to as Areafe:

$$Areafe=\frac{Area_{t_{i}}}{Area_{tote}}\times 100\;\mathrm{in}\;\%$$

Similar to the distance and the calculation of the rate of the demineralisation along this distance, the same methods were conducted for the area to determine the rate of the demineralisation of the area in µm^2^·s^−1^. Two rates were determined: the rate of the absolute area referred to as Arearate, which was conducted using the time reference $t_{0}$:

$$Arearate=\frac{Area_{t_{i}}-Area_{t_{0}}}{t_{i}-t_{0}}\mathrm{in}\;\text{µ}m^{2}\cdot s^{-1}$$

and the ‘area cumulative’ referred to as Areacrate (which detailed how fast a new demineralised region $t_{i}$ per time point evolved compared to the previous region $t_{\left(i-1\right)}$:

$$\;Areacrate=\frac{Area_{t_{i}}-Area_{t_{\left(i-1\right)}}}{t_{i}-t_{\left(i-1\right)}}\;\mathrm{in}\;\text{µ}m^{2}\cdot s^{-1}$$

- The evolution of the demineralised volume was also studied with visualisation and analysis. These measurements were similar to the area. The ratio of the volume of the demineralised region at time $t_{i}$, $V_{t_{i}}$ and the initial demineralised volume at the time reference $t_{0}$, $V_{t_{0}}$, were referred to as Vf:
  $$Vf=\frac{V_{t_{i}}}{V_{t_{0}}}$$

The ‘volume absolute’ from the reference time $t_{0}$ was referred to as Vol%:

$$Vol\%=\frac{V_{t_{i}}-V_{t_{0}}}{V_{t_{i}}}\times 100\;\mathrm{in}\;\%$$

The volume fraction of the demineralised region with the volume of enamel per time point $t_{i}$, was referred to as Vfe:

$$Vfe=\frac{V_{t_{i}}}{V_{tote}}$$

The rate of the progression of the demineralised volume was extracted from the time point analysed and was conducted for two rates, ‘volume rate absolute’, which were determined from the initial demineralised volume and the volume at a different time point, referred to as Vrateabs:

$$Vrateabs=\frac{V_{t_{i}}-V_{t_{0}}}{t_{i}-t_{0}}\text{µ}m^{3}\cdot s^{-1}$$

Additionally, the rate of the demineralisation per time point Vcrate, related to the ‘volume rate cumulative’:

$$Vcrate=\frac{V_{t_{i}}-V_{t\left(i-1\right)}}{t_{i}-t_{\left(i-1\right)}}\text{µ}m^{3}\cdot s^{-1}$$

The binary dataset in 2D and 3D of the demineralised region was carried out with Avizo, and the area, rate, and distance were analysed along with the plot of the Matlab and OriginPro software.

## 3. Results—Discussion

### 3.1. AFM 

**Figure 1:**
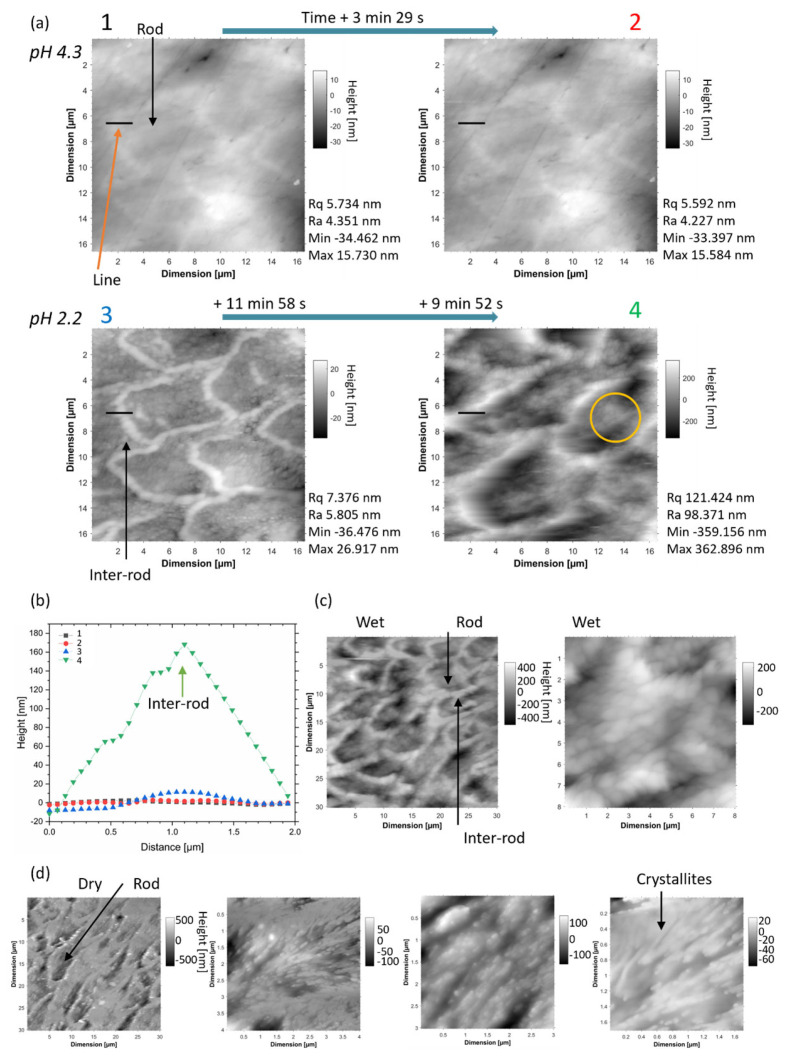
In situ AFM analysis of human enamel. (**a**) Time-lapse of AFM images showing the AFM height profiles of enamel during exposure to different pH 4.3 and 2.2, and the measurement of Rq, Ra and the minimum and maximum of the height reported. (**b**) Height line profile analysis from the locations in (**a**) highlighted with orange arrow on the first image, and (**c**) AFM analysis at a lower and higher magnification in a wet condition, and (**d**) in a dry condition.

Figure 1a shows a time-lapse of the AFM height profiles of demineralised enamel with pH 4.3 and 2.2. The rod shape in the images could be visualised before the demineralisation carried out here due to the presence of rod boundaries which were revealed upon fine polishing, as shown by SEM imaging (Supplementary Materials Figure S4). During demineralisation, the AFM height profile of the sample changed, and from the overall measurement, the rod demineralised faster than the inter-rod, as seen from the line profile extracted along the boundary. However, in the rods, heterogeneous regions could be identified, as highlighted in Figure 1 (additional details on surface analysis in Supplementary Materials Table S1). This experiment showed the onset of demineralisation, which, in the initial time frame, could not be obtained with tomography. However, the region analysed in the AFM was small. It was also important to take into consideration the shape of the tip in comparison with the crystallite organization [61]. The height profile in locations where the crystallites were packed may have been underestimated as the tip is not able to reach the deepest height. This onset of demineralisation was identified with AFM analysis at different time points after stopping immersion in acid [32]. Figure 1b shows images of the enamel after demineralisation at different scales in a wet environment and after drying. On a larger scale, the rod shape was visualised from the remaining boundary. 

### 3.2. Synchrotron Tomography Data

#### 3.2.1. Projection

From the raw data of the tomography analysis, enamel demineralisation was visible. The projections of a few datasets from the tomography acquisition are detailed in Supplementary Materials Figure S5 after the dark field and flat field correction. Zooming into a region at those time points is detailed in Supplementary Materials Figure S6. A line profile on the last time point highlights the features and variation in greyscale and reveals the rod dimensions (Supplementary Materials Figure S5c). The demineralisation region can be seen as a modification of image contrast with the formation of channels, similar to previous descriptions [8]. Interestingly, no significant changes, in contrast, were seen in the normal regions of enamel. On the projection images, the presence of bubbles was found, which interfered with the quality of the reconstruction; however, structural details could still be observed and analysed. These bubbles, as previously reported [26], were suggested to occur from the interaction of the X-ray beam with the water in the solutions used. 

#### 3.2.2. 2D Analysis of the Tomography Data

**Figure 2:**
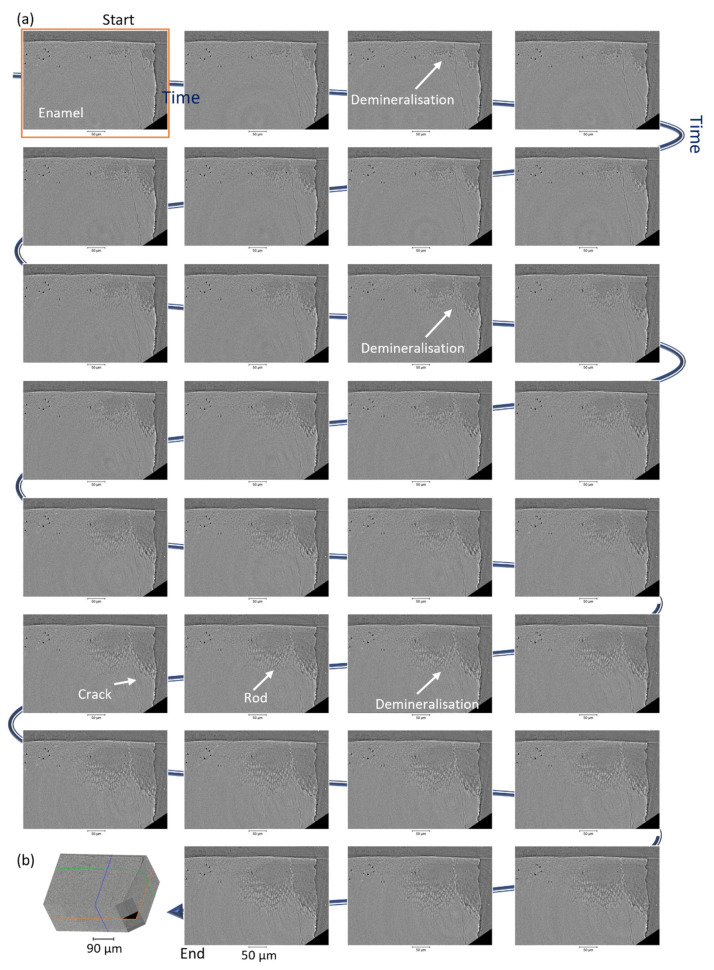
Illustration of the progression of the dissolution from one virtual slice of each time point tomogram. (**a**) Virtual slices in xy axis, from the first time point $t_{0}$ to the last time point $t_{end}$ (scale bar 50 µm), and (**b**) 3D view with the highlight of the plane displayed, data non-filtered, additional slices from the other axis in Supplementary Materials Figures S7 and S8. The varnish was also visualised on the virtual slices.

Virtual slices of the reconstructed data are shown in Figure 2 for the xy orientation and Supplementary Materials Figures S7 and S8 for the other two orientations (their locations are shown in Figure 2b) with the enamel structure that could be resolved as well as cracks. These images allow for the visualisation and evolution of the demineralisation with time and an increase in the dark regions (corresponding with the loss of material) which indicated a decrease in density (Beer-Lambert law [62]). Those changes appeared to be non-homogenous—anisotropic; this is highlighted in Figure 2 and Supplementary Materials Figure S9 with two slices from a one-time frame, and the view of the demineralisation and modification of the structure, based on the assignment of the rod and inter-rod regions. In the non-demineralised region, the enamel structure rods and inter-rods were not visualised using this setup, as noticed in a previous study from another setup [6]. These datasets, at each time point, were rich in information, and demineralisation was apparent in each reconstructed dataset. A more mineralised region could be seen at the surface of the enamel (which was also found in non-cavitated carious enamel lesions) and referred to as region 1 or the surface zone [63,64]. The analysis of this region was detailed in Supplementary Materials Figure S10 using three-line grey value profiles and a 3D view. The change in the grey value of the slices with time provided information on the dynamic gradual changes during dissolution, using an operando analysis, and no interruption on the exposure of enamel to the acid was necessary [65]. 

With progression in time, it was possible to visualise the rods and inter-rod with high resolution, and thus, time points could be processed (see Supplementary Materials Movie S1 with time-lapse of the virtual slices) to highlight the dynamic changes in the structure and in parallel with the acquisition of the data.

#### 3.2.3. 3D Analysis and the Addition of Time

**Figure 3:**
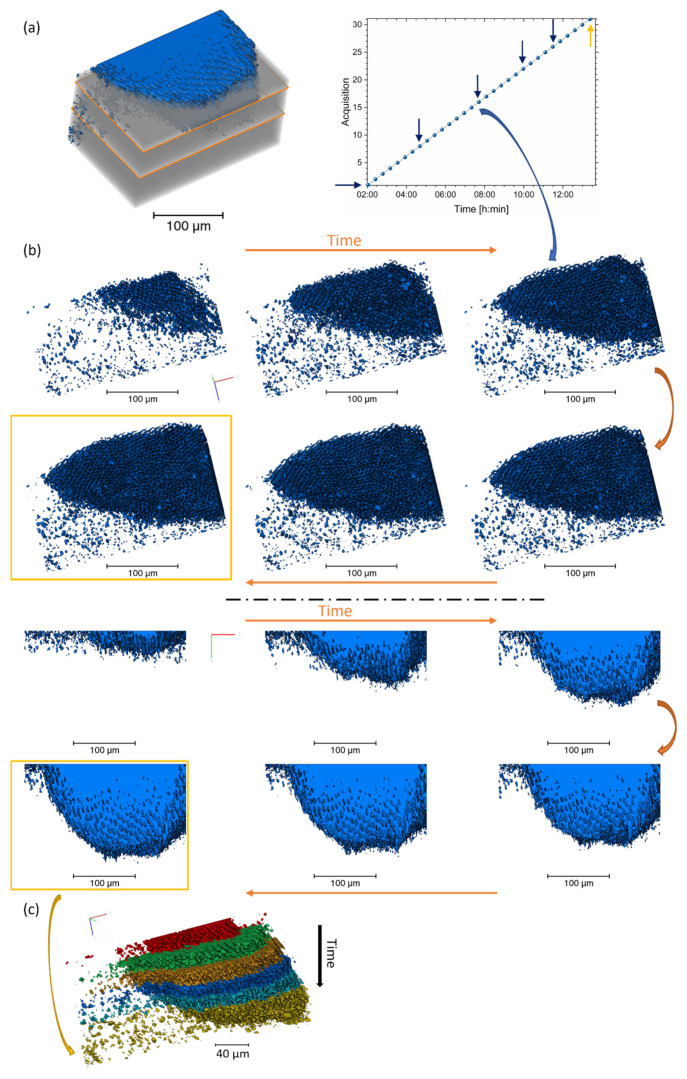
3D renderings of the segmented region of several datasets. (**a**) Illustration of the rendering of the volume of enamel (1280 × 871 × 550 pixels) and background with an overlap of the segmented region from the region of interest 812 × 514 × 410 pixels (263.575 × 166.725 × 132.925 µm, voxel size 0.325 µm) for the data $t_{7}$ (last dataset). A plot of the dataset with time, where arrows highlight the time points illustrated in Figure. (**b**,**c**) A sequence of 3D rendering of the segmented dataset with time located in (**a**) with different orientations. See Supplementary Materials Figure S12 for more details on the region segmented. (**c**) 3D renderings of the different time points illustrating the progression of the demineralisation and the colour assigned to the time points. Additional details of the volumes are in Supplementary Materials Movies S2 and S3.

A 4D visualisation and analysis of the dataset were carried out on the segmented region across several time points, as shown in Figure 3. All the analysed tomograms showed a demineralised region in the enamel structure. A variation in the volume and progression of the demineralisation could be seen with time. It was found that at the advanced front, the inter-rod substance was preferentially demineralised in comparison with the rods. This can be seen in Figure 3, Supplementary Materials Figures S11 and S12. These findings were different from the ones found in AFM; however, there were differences in the analysis. In the AFM, the sample was polished and already had surface modification, and the analysis was based on the surface and on the initial time of demineralisation. In comparison to the tomography data, in this one, the sample was not polished, and the image analysis tracked the modifications after a long period of demineralisation without the start of the modification of the structure from the 3D analysis. This could explain the disparities in the results of the demineralisation seen, and more studies are required to evaluate these changes.

**Figure 4:**
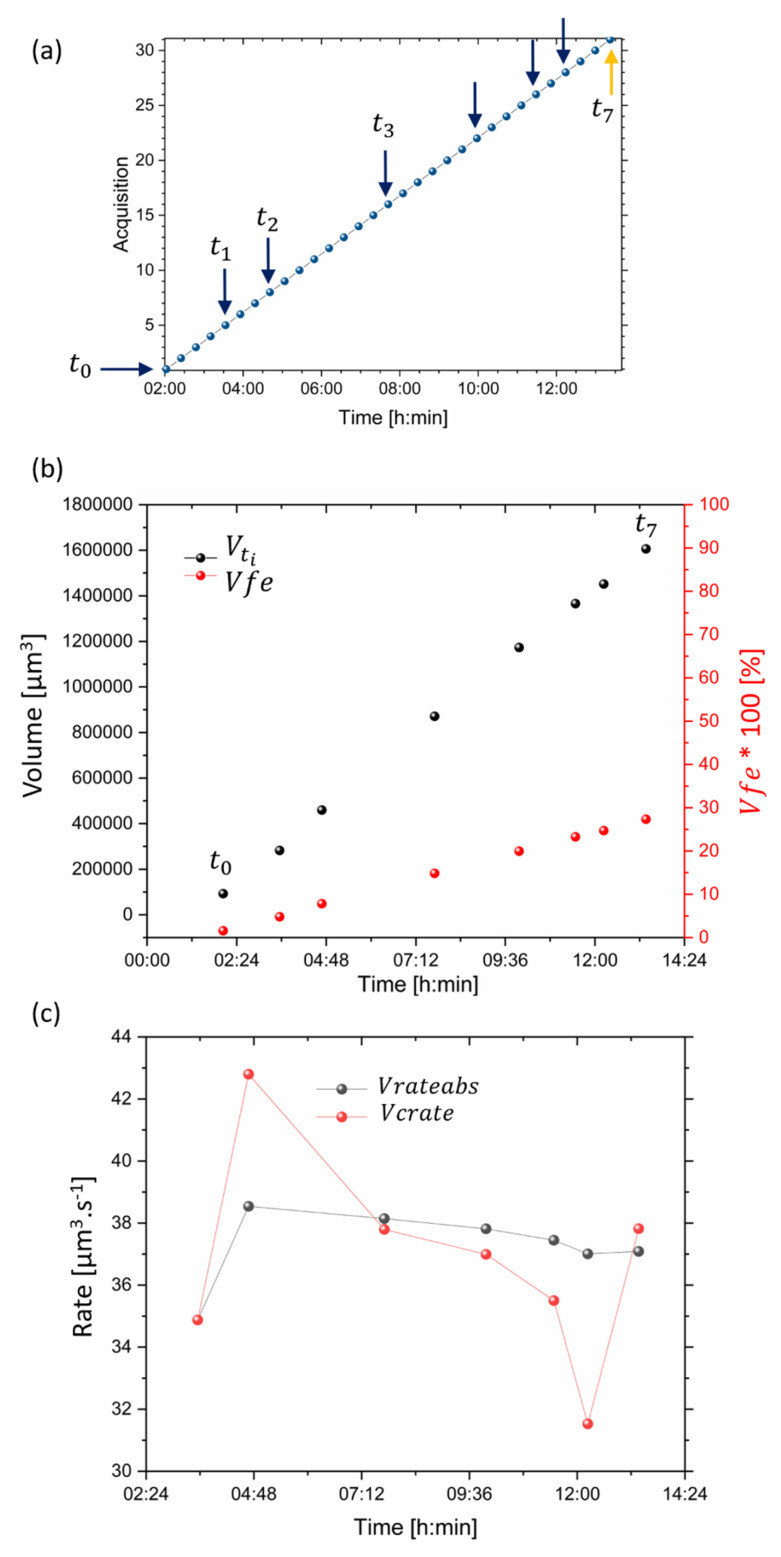
Analysis of the volume of the demineralised regions of several datasets with time. (**a**) A plot of the dataset with time, where arrows highlight the time points illustrated in Figure. (**b**) The volume of the segmented region at each time point $V_{t_{i}}$ (in black), and ratio with the total volume of the enamel highlighting the evolution of the fraction of demineralised region per time point (in red). (**c**) A plot of the rate of the cumulative (in red) and absolute volume (in black) showing the variation in the rate of demineralisation with time.

A pattern in the rod structure (HSBs suggested) was also apparent along the depth of the volume (Supplementary Materials Movie S4 with the details of one dataset). This structure was only revealed after demineralisation took place. The progression of the lesion volume with time was plotted (Figure 4) to reveal the progression of demineralisation. In addition, the cumulative volume was determined, as well as the rate of demineralisation, as shown in Figure 4c, where the rate was found to be non-linear and was visualised from the variation in rate around 4 h and 12 h. Supplementary Materials Figures S11 and S13–S15 showed the demineralisation pathways around the rods and striations, highlighting the progression of the demineralisation of the enamel structure.

In addition to the visualisation of the demineralised region, Euclidean maps were determined from a few regions in the enamel, showing differences in the remaining enamel in and outside the lesion in Supplementary Materials Figure S16. A small region was analysed to visualise the progression of the lesion along a few rods (detailed Supplementary Materials Figure S17) and showed the anisotropy in the progression of the lesion between two-time points $t_{3}$ and $t_{7}$, confirming the necessity to perform 3D analysis and not only projections to provide the progression of the lesions with high spatial and resolution details.

### 3.3. Area Evolution

**Figure 5:**
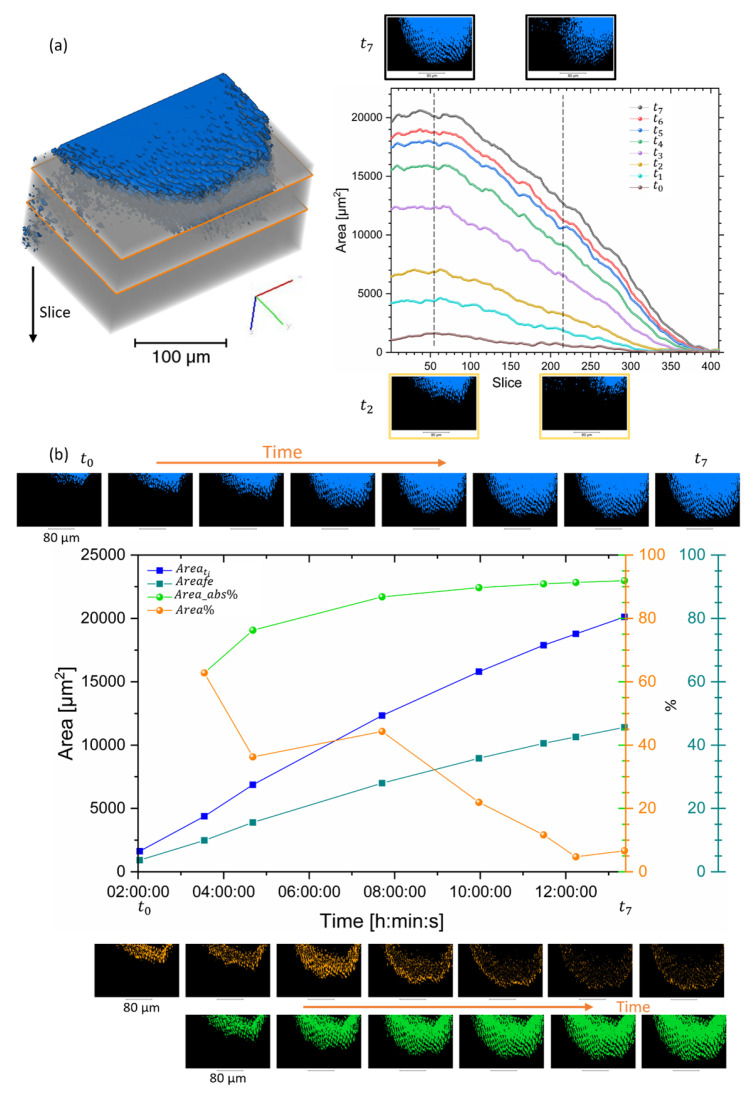
Analysis of the area of the demineralised region in several datasets and with time. (**a**) Volume rendering of the dataset $t_{7}$ with two virtual slices and the plot of the evolution of the area (xy plane) and the slice corresponding with the depth of the sample along z axis, and the thickness of each slice 0.325 µm. Illustration of a few slices from $t_{2}$ and $t_{7}$ (scale bar 80 µm). The time points are detailed in Figure 4a. (**b**) A plot of the total area, the Areafe, and progression of the area with time for one slice extracted in the different datasets highlights the variation in the evolution of the demineralisation. Illustration of the region analysed; in blue is the total area, in orange is the difference between consecutive increments and green represents the difference from $t_{0}$, see methods section for additional details.

**Figure 6:**
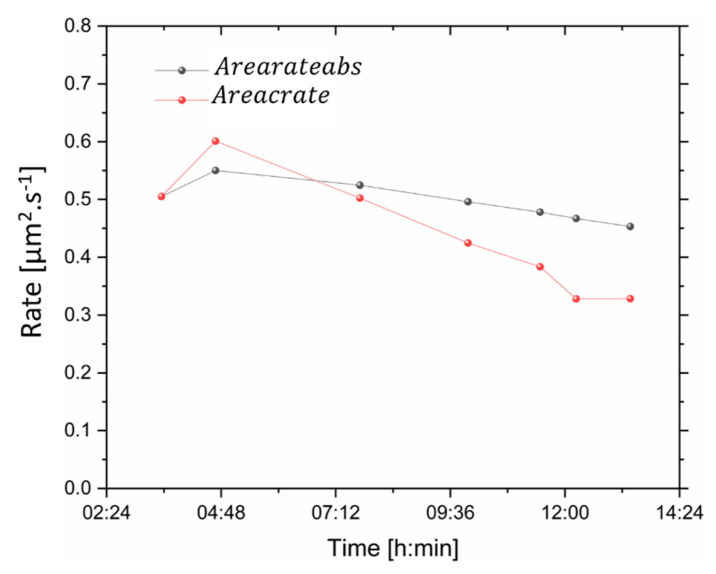
Analysis of the area of demineralised regions with time from one plane xy. A plot of the rate of the area accumulative and in comparison, with the initial dataset showing evolution with time. The time points are detailed in Figure 4a.

The evolution of the area of the demineralisation regions at each time point and plane location in the dataset was investigated. The evolution of the segmented lesion in different datasets clearly showed the evolution of the dissolution (Figure 5) with time. This could be carried out on each virtual slice, where Figure 5 displays some of them, and the locations of the slices were highlighted on the 3D rendering of the enamel. The progression of the area was found to be non-linear. A decrease in the area was found with the depth in the sample (Figure 5a), which approached the border of the demineralised region. The visualisation of the demineralisation process was important, but this could be extended further by the quantification of the process. The demineralised area was extracted in the dataset at different time points, and Figure 5b details the progression of the demineralised area with time with an accumulated and absolute measurement. The measurement of the area was carried out in many datasets to extract a representative slice (Figure 5b). Overall, a decrease in the progression of the demineralised area with time was seen with the drop in the cumulative area and a drop in the progression of Areaf with time. This provided information on the demineralisation but missed the 3D progression of the demineralisation, as seen in the Volume section of this manuscript. From the computation of the area, it was possible then to extract the rates of demineralisation, which were non-linear and varied between different slices, Figure 6. 

### 3.4. Distance Evolution

**Figure 7:**
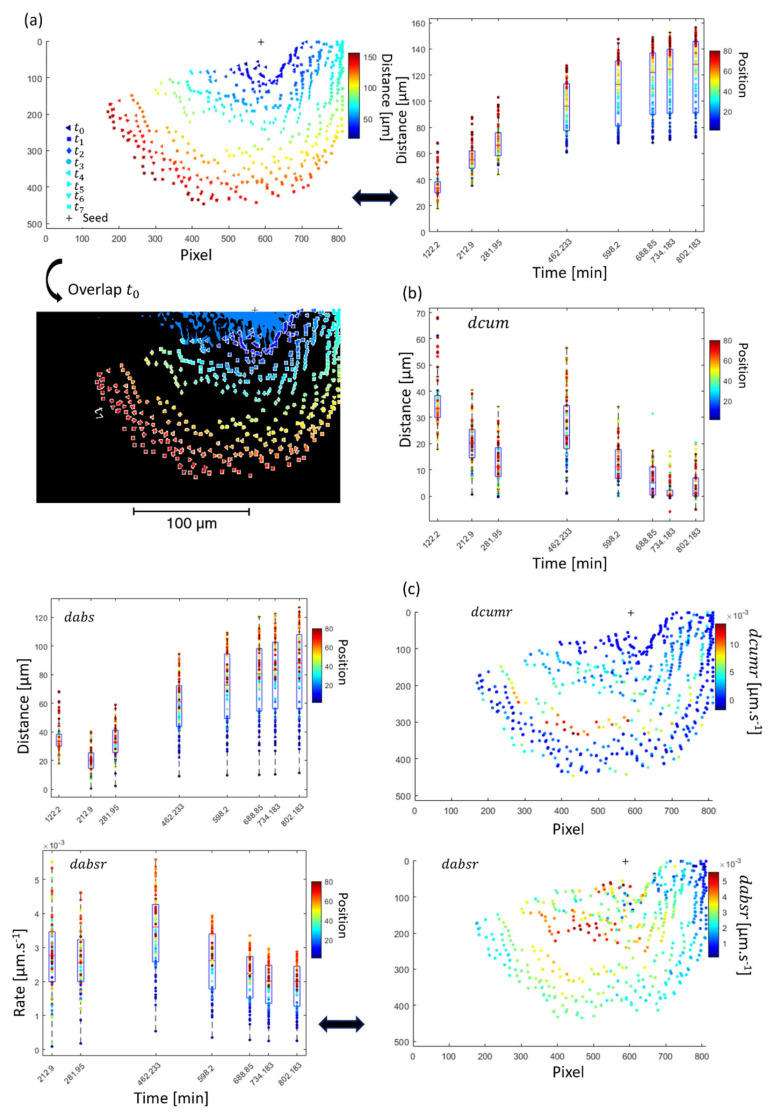
Progression in terms of spatial analysis with the farthest coordinate in many locations of the segmented regions. (**a**) Analysis of the distance with statistics and distance dabs and distance dcum in (**b**). (**c**) Rate details at different locations and times. For increment 7 equivalent to $t_{7}$ − $t_{6}$ in terms of duration (first one being $t_{0}$, the details of the datasets used are described in Figure 4a). On a first indication, a similar approach could be carried on the projection but was not carried out here because the reconstructed virtual slices were analysed.

The 3D reconstruction also enabled the extraction of 1D profiles. Thus, the distance of the demineralisation region was measured from one point on the slice (equivalent to a seed) to the front of the lesion on the same virtual slice and along different angles (Figure 7 and Figure S3), which was then carried out on the different time point. The measurement of the distance of demineralisation at various angles showed the anisotropy of the dissolution in a high resolution in the structure with different distances and times. Figure 7 details the measurements of the distance of the demineralisation with location and time. It can be observed that there were differences in the progression of the lesion at different locations in the structure, Figure 7a,b. The distance of the front demineralisation increased with time but non-linearly. This allowed the demineralisation to be tracked. The rates from the distance cumulated and absolute were also extracted, and inhomogeneity was found (Figure 7c). Localised rates were calculated at different locations in the 3D reconstruction of the sample. The maximum rate was found to be ~5.58 nm·s^−1^ in the region analysed, and a variation in the average rate was visualised. The rate calculated could be compared with previous studies containing enamel and is summarised in Table 1. There were variations in the rate found, which could be explained for several reasons, including differences in the setup, the sample, pH, and the time of demineralisation. It also highlighted the importance of visualising and analysing the structure to have more details on these rates due to the inhomogeneity of the demineralisation, as well as the difficulty in providing only one value for the rate for the line analysis. In comparison with the remineralisation study of a recent study demonstrating the remineralisation of enamel using biomimetic mineralisation, the rate of remineralisation could be 400 times less in order of magnitude compared with the demineralisation process. This indicated the difficulty of reversing the demineralisation process in terms of speed but also in terms of location. 

This study showed the feasibility of analysing the demineralisation of enamel with fast acquisition and high resolution. This provided insight into the analysis of enamel caries and should be transferred to additional demineralised and remineralised studies. Based on this study, it was suggested that enamel could be further characterised with this technique and using alternating processes of remineralisation and demineralisation occurring in the oral environment.

## 4. Conclusions

This study shows the feasibility of the in situ analysis of enamel with high spatial and temporal resolutions to better understand the dynamic process of enamel dissolution. 

Using X-ray tomography in a fluidic setup, it was found that the rate of demineralisation varied at different locations in enamel with temporal changes. The 3D reconstructed data enabled the comprehensive analysis of the rate of demineralisation ranging from 1D to 3D. Furthermore, the visualisation of the structural changes across different regions confirmed the inhomogeneity of the demineralisation. With the use of in situ AFM imaging, structural changes could be monitored at the nanoscale with the advantage of being able to identify the onset of demineralisation. 

Overall, this correlative imaging technique opened up the possibility of understanding dynamic processes from 1D to 4D under different conditions, e.g., pH, flow rate and different demineralisation and remineralisation solutions. This could be further combined with other analytical techniques, e.g., WAXS, for crystallographic information such as crystal orientation and dimension.

## Figures and Tables

**Table 1:** Studies of the rate of demineralisation and remineralisation of enamel. The table summarises some studies on the artificial demineralisation of enamel with the condition used, the rate measured and a comparison with a remineralised study.

Technique	pH—Condition	Rate	Measurement	Reference
Tomography	2.2 lactic acid	0.54–5.58 nm·s^−1^ (see Figure 7)	Line profile after ~7.7 h	This study
Tomography	2.2 lactic acid	38.14 × 10^10^ nm^3^·s^−1^ (see Figure 4)	Volume after ~7 h 40 min	This study
SAXS	2.2 lactic acid	1.4 nm·s^−1^	Line profile	[38]
Radiography	2.2 lactic acid	3.4 nm·s^−1^	Line profile longest distance after 85 h	[31]
Tomography	4 acetic acid	1.45 × 10^13^ nm^3^·s^−1^	Volume after 6 days	[51]
Tomography	4 acetic acid	1.5 nm·s^−1^	Distance after 6 days	[51]
SEM	Remineralisation	0.015 nm·s^−1^	Distance	[66]

## Data Availability

Data collected and interpreted in this study is maintained by the authors and can be made available upon request.

## Supplementary Materials

The following supporting information can be downloaded at: https://www.mdpi.com/article/10.3390/dj11050130/s1, Table S1, Figures S1–17 and Movies S1–S4.

## References

[1] James S.L., Abate D., Abate K.H., Abay S.M., Abbafati C., Abbasi N., Abbastabar H., Abd-Allah F., Abdela J., Abdelalim A. (2018). Global, regional, and national incidence, prevalence, and years lived with disability for 354 diseases and injuries for 195 countries and territories, 1990–2017: A systematic analysis for the Global Burden of Disease Study 2017. Lancet.

[2] Fejerskov O., Kidd E.A.M., Nyvad B., Baelum V., Fejerskov O., Kidd E.A.M. (2008). Defining the disease: An introduction. Dental Caries the Disease and Its Clinical Management.

[3] Forssten S.D., Björklund M., Ouwehand A.C. (2010). Streptococcus mutans, Caries and Simulation Models. Nutrients.

[4] Hwang G., Liu Y., Kim D., Sun V., Aviles-Reyes A., Kajfasz J.K., Lemos J.A., Koo H. (2016). Simultaneous spatiotemporal mapping of in situ pH and bacterial activity within an intact 3D microcolony structure. Sci. Rep..

[5] Marsh P.D., Moter A., Devine D.A. (2011). Dental plaque biofilms: Communities, conflict and control. Periodontology 2000.

[6] Besnard C., Marie A., Buček P., Sasidharan S., Harper R.A., Marathe S., Wanelik K., Landini G., Shelton R.M., Korsunsky A.M. (2022). Hierarchical 2D to 3D micro/nano-histology of human dental caries lesions using light, X-ray and electron microscopy. Mater. Des..

[7] Voegel J.C., Frank R.M. (1977). Stages in the dissolution of human enamel crystals in dental caries. Calcif. Tissue Int..

[8] Besnard C., Harper R.A., Moxham T.E.J., James J.D., Storm M., Salvati E., Landini G., Shelton R.M., Korsunsky A.M. (2021). 3D analysis of enamel demineralisation in human dental caries using high-resolution, large field of view synchrotron X-ray micro-computed tomography. Mater. Today Commun..

[9] Salvati E., Besnard C., Harper R.A., Moxham T., Shelton R.M., Landini G., Korsunsky A.M. (2021). Finite Element Modelling and Experimental Validation of the Enamel Demineralisation Process at the Rod Level. J. Adv. Res..

[10] Besnard C., Harper R.A., Salvati E., Moxham T.E.J., Romano Brandt L.R., Landini G., Shelton R.M., Korsunsky A.M. (2021). Analysis of in vitro demineralised human enamel using multi-scale correlative optical and scanning electron microscopy, and high-resolution synchrotron wide-angle X-ray scattering. Mater. Des..

[11] Besnard C., Marie A., Sasidharan S., Harper R.A., Shelton R.M., Landini G., Korsunsky A.M. (2023). Synchrotron X-ray Studies of the Structural and Functional Hierarchies in Mineralised Human Dental Enamel: A State-of-the-Art Review. Dent. J..

[12] Besnard C., Marie A., Buček P., Sasidharan S., Harper R.A., Marathe S., Wanelik K., Landini G., Shelton R.M., Korsunsky A.M. (2022). Movies and dataset for: Hierarchical 2D to 3D micro/nano-histology of human dental caries lesions using light, X-ray and electron microscopy. Mendeley Data.

[13] Featherstone J.D.B., Lussi A. (2006). Understanding the Chemistry of Dental Erosion. Monogr. Oral Sci..

[14] Bonte E., Deschamps N., Goldberg M., Vernois V. (1988). Quantification of Free Water in Human Dental Enamel. J. Dent. Res..

[15] Daculsi G., Menanteau J., Kerebel L.M., Mitre D. (1984). Length and shape of enamel crystals. Calcif. Tissue Int..

[16] DeRocher K.A., Smeets P.J.M., Goodge B.H., Zachman M.J., Balachandran P.V., Stegbauer L., Cohen M.J., Gordon L.M., Rondinelli J.M., Kourkoutis L.F. (2020). Chemical gradients in human enamel crystallites. Nature.

[17] Beniash E., Stifler C.A., Sun C.-Y., Jung G.S., Qin Z., Buehler M.J., Gilbert P.U.P.A. (2019). The hidden structure of human enamel. Nat. Commun..

[18] Besnard C., Marie A., Sasidharan S., Buček P., Walker J.M., Parker J.E., Moxham T.E.J., Daurer B., Kaulich B., Kazemian M. (2022). Nanoscale correlative X-ray spectroscopy and ptychography of carious dental enamel. Mater. Des..

[19] Risnes S., Saeed M., Sehic A., Papagerakis P. (2019). Scanning Electron Microscopy (SEM) Methods for Dental Enamel. Odontogenesis: Methods and Protocols.

[20] Cui F.-Z., Ge J. (2007). New observations of the hierarchical structure of human enamel, from nanoscale to microscale. J. Tissue Eng. Regen. Med..

[21] Kelly A.M., Kallistova A., Küchler E.C., Romanos H.F., Lips A., Costa M.C., Modesto A., Vieira A.R. (2020). Measuring the Microscopic Structures of Human Dental Enamel Can Predict Caries Experience. J. Pers. Med..

[22] Lynch C.D., O’sullivan V.R., Dockery P., McGillycuddy C.T., Sloan A.J. (2010). Hunter-Schreger Band patterns in human tooth enamel. J. Anat..

[23] Risnes S. (1985). A scanning electron microscope study of the three-dimensional extent of Retzius lines in human dental enamel. Eur. J. Oral Sci..

[24] Yun F., Swain M.V., Chen H., Cairney J., Qu J., Sha G., Liu H., Ringer S.P., Han Y., Liu L. (2020). Nanoscale pathways for human tooth decay–Central planar defect, organic-rich precipitate and high-angle grain boundary. Biomaterials.

[25] Korsunsky A.M., Besnard C., Marie A., Sasidharan S., Harper R.A., James J.D., Landini G., Shelton R.M., Marathe S. Time-resolved operando X-ray micro-computed tomography of the demineralisation of human dental enamel. Proceedings of the ESRF User Meeting.

[26] Leung N., Harper R.A., Zhu B., Shelton R.M., Landini G., Sui T. (2021). 4D microstructural changes in dentinal tubules during acid demineralisation. Dent. Mater..

[27] Dawes C. (2003). What is the critical pH and why does a tooth dissolve in acid?. J. Can. Dent. Assoc..

[28] Risnes S., Li C. (2019). On the method of revealing enamel structure by acid etching. Aspects of optimization and interpretation. Microsc. Res. Tech..

[29] Poggio C., Ceci M., Beltrami R., Lombardini M., Colombo M. (2014). Atomic force microscopy study of enamel remineralization. Ann. Stomatol..

[30] Scott D.B., Simmelink J.W., Nygaard V. (1974). Structural Aspects of Dental Caries. J. Dent. Res..

[31] Harper R.A., Shelton R.M., James J.D., Salvati E., Besnard C., Korsunsky A.M., Landini G. (2021). Acid-induced demineralisation of human enamel as a function of time and pH observed using X-ray and polarised light imaging. Acta Biomater..

[32] Li P., Oh C., Kim H., Chen-Glasser M., Park G., Jetybayeva A., Yeom J., Kim H., Ryu J., Hong S. (2020). Nanoscale effects of beverages on enamel surface of human teeth: An atomic force microscopy study. J. Mech. Behav. Biomed. Mater..

[33] Parkinson C.R., Shahzad A., Rees G.D. (2010). Initial stages of enamel erosion: An in situ atomic force microscopy study. J. Struct. Biol..

[34] Pyne A., Marks W., Picco L.M., Dunton P.G., Ulcinas A., Barbour M.E., Jones S.B., Gimzewski J., Miles M.J. (2009). High-speed atomic force microscopy of dental enamel dissolution in citric acid. Arch. Histol. Cytol..

[35] Wang L., Tang R., Bonstein T., Orme C.A., Bush P.J., Nancollas G.H. (2005). A New Model for Nanoscale Enamel Dissolution. J. Phys. Chem. B.

[36] Watari F. (2005). In situ quantitative analysis of etching process of human teeth by atomic force microscopy. J. Electron Microsc..

[37] Liu B.H., Yu L.-C. (2017). In-situ, time-lapse study of extracellular polymeric substance discharge in Streptococcus mutans biofilm. Coll. Surf. B Biointerfaces.

[38] Sui T., Salvati E., Harper R.A., Zhang H., Shelton R.M., Landini G., Korsunsky A.M. (2018). In situ monitoring and analysis of enamel demineralisation using synchrotron X-ray scattering. Acta Biomater..

[39] Voltolini M., Haboub A., Dou S., Kwon T.-H., MacDowell A.A., Parkinson D.Y., Ajo-Franklin J. (2017). The emerging role of 4D synchrotron X-ray micro-tomography for climate and fossil energy studies: Five experiments showing the present capabilities at beamline 8.3.2 at the Advanced Light Source. J. Synchrotron Radiat..

[40] Ni X., Fritz N.K., Wardle B.L. (2019). In Situ Testing Using Synchrotron Radiation Computed Tomography in Materials Research. MRS Adv..

[41] Fernández M.P., Barber A.H., Blunn G.W., Tozzi G. (2018). Optimization of digital volume correlation computation in SR-microCT images of trabecular bone and bone-biomaterial systems. J. Microsc..

[42] Cai B., Wang J., Kao A., Pericleous K., Phillion A.B., Atwood R.C., Lee P.D. (2016). 4D synchrotron X-ray tomographic quantification of the transition from cellular to dendrite growth during directional solidification. Acta Mater..

[43] Shuai S., Guo E., Phillion A.B., Callaghan M.D., Jing T., Lee P.D. (2016). Fast synchrotron X-ray tomographic quantification of dendrite evolution during the solidification of Mg Sn alloys. Acta Mater..

[44] Zhang Z., Wang C., Koe B., Schlepütz C.M., Irvine S., Mi J. (2021). Synchrotron X-ray imaging and ultrafast tomography in situ study of the fragmentation and growth dynamics of dendritic microstructures in solidification under ultrasound. Acta Mater..

[45] Guo E., Zeng G., Kazantsev D., Rockett P., Bent J., Kirkland M., Van Dalen G., Eastwood D.S., StJohn D., Lee P.D. (2017). Synchrotron X-ray tomographic quantification of microstructural evolution in ice cream–a multi-phase soft solid. RSC Adv..

[46] Polacci M., Arzilli F., La Spina G., Le Gall N., Cai B., Hartley M.E., Di Genova D., Vo N.T., Nonni S., Atwood R.C. (2018). Crystallisation in basaltic magmas revealed via in situ 4D synchrotron X-ray microtomography. Sci. Rep..

[47] Hao S., Daemi S.R., Heenan T.M.M., Du W., Tan C., Storm M., Rau C., Brett D.J.L., Shearing P.R. (2021). Tracking lithium penetration in solid electrolytes in 3D by in-situ synchrotron X-ray computed tomography. Nano Energy.

[48] Marone F., Schlepütz C.M., Marti S., Fusseis F., Velásquez-Parra A., Griffa M., Jiménez-Martínez J., Dobson K.J., Stampanoni M. (2020). Time Resolved in situ X-Ray Tomographic Microscopy Unraveling Dynamic Processes in Geologic Systems. Front. Earth Sci..

[49] Singh S.S., Williams J.J., Stannard T.J., Xiao X., De Carlo F., Chawla N. (2016). Measurement of localized corrosion rates at inclusion particles in AA7075 by in situ three dimensional (3D) X-ray synchrotron tomography. Corros. Sci..

[50] Davis G., Müller B., Wang G. (2019). Time-lapse x-ray microtomography for detecting small changes in local mineral concentration. SPIE Optical Engineering + Applications-Developments in X-ray Tomography XII.

[51] Davis G.R., Mills D., Anderson P. (2018). Real-time observations of tooth demineralization in 3 dimensions using X-ray microtomography. J. Dent..

[52] Dowker S.E.P., Elliott J.C., Davis G.R., Wassif H.S. (2003). Longitudinal Study of the Three-Dimensional Development of Subsurface Enamel Lesions during in vitro Demineralisation. Caries Res..

[53] Featherstone J.D.B., O’Reilly M.M., Shariati M., Brugler S., Leach S.A. (1985). Enhancement of remineralisation in vitro and in vivo. Proceedings of the Factors Relating to Demineralisation and Remineralisation of the Teeth: Proceedings of a Workshop October 5–10, 1985.

[54] Featherstone J.D.B., Shariati M., Brugler S., Fu J., White D.J. (1988). Effect of an Anticalculus Dentifrice on Lesion Progression under pH Cycling Conditions in vitro. Caries Res..

[55] Rau C., Bodey A., Storm M., Cipiccia S., Marathe S., Zdora M.-C., Zanette I., Wagner U., Batey D., Shi S., Müller B., Wang G. (2017). Micro- and nano-tomography at the DIAMOND beamline I13L imaging and coherence. SPIE Optical Engineering + Applications-Developments in X-ray Tomography XI..

[56] Rau C., Wagner U., Pešić Z., De Fanis A. (2011). Coherent imaging at the Diamond beamline I13. Phys. Status Solidi (a).

[57] Eisenburger M., Addy M., Hughes J.A., Shellis R.P. (2001). Effect of Time on the Remineralisation of Enamel by Synthetic Saliva after Citric Acid Erosion. Caries Res..

[58] Scientific T. (2019). User’s Guide Avizo Software 2019.

[59] Rasband W.S. (2018). ImageJ..

[60] Schindelin J., Arganda-Carreras I., Frise E., Kaynig V., Longair M., Pietzsch T., Preibisch S., Rueden C., Saalfeld S., Schmid B. (2012). Fiji: An open-source platform for biological-image analysis. Nat. Methods.

[61] Vitkov L., Kastner M., Kienberger F., Hinterdorfer P., Schilcher K., Grunert I., Dumfahrt H., Krautgartner W.D. (2008). Correlations between AFM and SEM Imaging of Acid-Etched Tooth Enamel. Ultrastruct. Pathol..

[62] Kastner J., Heinzl C., Ida N., Meyendorf N. (2018). X-ray tomography. Handbook of Advanced Non-Destructive Evaluation.

[63] Goldberg M., Goldberg M. (2016). Enamel softening (dental erosion)-enamel etching-the early enamel carious lesion. Understanding Dental Caries.

[64] Goldberg M. (2017). Deciduous tooth and dental caries. Ann. Pediatr. Child Health.

[65] Shahani A.J., Xiao X., Lauridsen E.M., Voorhees P.W. (2020). Characterization of metals in four dimensions. Mater. Res. Lett..

[66] Shao C., Jin B., Mu Z., Lu H., Zhao Y., Wu Z., Yan L., Zhang Z., Zhou Y., Pan H. (2019). Repair of tooth enamel by a biomimetic mineralization frontier ensuring epitaxial growth. Sci. Adv..

