# Supplementary material for: Time-Lapse In Situ 3D Imaging Analysis of Human Enamel Demineralisation Using X-ray Synchrotron Tomography
Source: Dent J (Basel). 2023 May 9;11(5):130. doi: 10.3390/dj11050130 (PMC10217016; doi:10.3390/dj11050130)
Supplement: Supplementary file 1 [file dentistry-11-00130-s001.zip › Supplementaryinformation_cor_.pdf]

Time-lapse In Situ 3D Imaging Analysis of Human Enamel Demineralisation Using X-ray Synchrotron Tomography

Authors

Cyril Besnard*[1], Ali Marie[1*], Sisini Sasidharan[1], Robert A. Harper[2], Shashidhara Marathe[3], Jonathan Moffat[4], Richard M. Shelton[2], Gabriel Landini[2], Alexander M. Korsunsky[1]

[1] MBLEM, Department of Engineering Science, University of Oxford, Parks Road, Oxford, Oxfordshire, OX1 3PJ, U.K.

[2] School of Dentistry, University of Birmingham, 5 Mill Pool Way, Edgbaston, Birmingham, West Midlands, B5 7EG, U.K.

[3] Diamond Light Source Ltd., Didcot, Oxfordshire, OX11 0DE, U.K.

[4] Oxford Instruments Asylum Research, Halifax Rd, High Wycombe, Bucks HP12 3SE, U.K.

Email addresses:

cyril.besnard@eng.ox.ac.uk, ali.marie@eng.ox.ac.uk, sisini.sasidharan@eng.ox.ac.uk, R.A.Harper@bham.ac.uk, shashidhara.marathe@diamond.ac.uk, jonathan.moffat@oxinst.com, R.M.Shelton@bham.ac.uk, G.Landini@bham.ac.uk, alexander.korsunsky@eng.ox.ac.uk

* **Corresponding authors:**

Cyril Besnard, cyril.besnard@eng.ox.ac.uk

Ali Marie, dralism@gmail.com

Supplementary Information (SI)

Table of Contents

Table

Supplementary Table S1. pp. 3-8

Figures

Supplementary Fig. S1.	p. 9
Supplementary Fig. S2.	p. 10
Supplementary Fig. S3.	p. 11
Supplementary Fig. S4.	p. 12
Supplementary Fig. S5.	pp. 13-14
Supplementary Fig. S6.	p. 15
Supplementary Fig. S7.	pp. 16-17
Supplementary Fig. S8.	pp. 18-19
Supplementary Fig. S9.	p. 19
Supplementary Fig. S10.	p. 20
Supplementary Fig. S11.	pp. 21-22
Supplementary Fig. S12.	p. 23
Supplementary Fig. S13.	pp. 24-25
Supplementary Fig. S14.	pp. 26-27
Supplementary Fig. S15.	p. 28
Supplementary Fig. S16.	p. 29
Supplementary Fig. S17.	p. 30

Movies

Movies S1-S4. p. 31

Supplementary Table S1. Details about atomic force microscopy (AFM) and profilometry applications on dental research. Table with the review of references (Ref.) on AFM application on tooth, static and *in situ* experiments for humans and animals, as well as the details of profilometry applications.

For the AFM, measurement can be extended with mechanical properties using e.g. elastic modulus[1-3], and adhesion test to study bacteria adhesion[4], and interactions[5]. This highlighted the versatility of this equipment which is also used to understand the development of enamel and provided new insights on the formation[6] and maturation of enamel[7] and for remineralisation studies[8]. *In situ* experiments can also be transferred to other applications such as corrosion test[9].

Ref.	Human	Animal	Locations	Static	Time
10	x		Dentine, wet and drying		x
11	x		Dentine, distilled water and etched using citric acid, wet, desiccate and hydration condition		x
12,13	x		Dentine exposed to different agents, phosphoric acid, maleic acid, review	x	
14	x		Enamel etched using HCl and citric acid, indentation	x	
15	x		Enamel, intra-oral appliance, with and without exposure to different drinks	x	
16	x		Enamel, dentine, DEJ, nanoscratch	x	
17	x		Enamel exposed to different solutions of citric acid		
18	x		Enamel demineralised using citric acid, remineralised	x	
19	x		Enamel, intra-oral appliance, exposed to different drinks, remineralisation	x	
20	x	x	Enamel with and without demineralisation	x	
21	x		Enamel, hypoplasia, normal	x	
22	x		Enamel, dentine, etched with different acids		x
2	x		Enamel with and without etching using citric acid, indentation	x	
23	x		Dentine, transparent and normal	x	
24	x		Enamel with and without demineralisation using HCl		x

25	x		Enamel demineralised using lactic acid, remineralised	x	
26	x		Enamel	x	
27	x		Enamel exposed to orthophosphoric acid	x	
28	x		Enamel exposed to different solutions, citrus drinks, indentation	x	
29	x		Tooth from groups with down syndrome, cerebral palsy and control, enamel etched using citric acid	x	
30	x		Enamel etched using citric acid	x	
31	x		Enamel with and without treatment using different phosphoric acid solutions	x	
32	x		Enamel before and after bleaching	x	
33	x		Enamel exposed to citric acid, mouthrinse	x	
34	x		Enamel and dentine, healthy and urenic groups, with and without bleaching	x	
35		x	Enamel with and without fluoride treatment demineralised using citric acid	x	x
36	x		Enamel with and without fluoride treatment, etching using phosphoric acid	x	
37	x		Section of tooth	x	
38	x		Enamel with and without exposition to hydrogen peroxide	x	
39	x		Enamel with and without laser treatments	x	
40	x		Enamel before and after demineralisation using phosphoric acid and remineralisation	x	
41	x		Enamel	x	

42	x		Enamel with and without acidulated phosphate fluoride treatment, etched using phosphoric acid	x	
43	x		Enamel and dentine, groups with chronic renal failure, with and without etching using phosphoric acid etchant		
44	x		Enamel treated with different bleaching agents and control	x	
45	x		Enamel without and without treatment using phosphoric acid, pumiced enamel	x	
46	x		Enamel with and without treatment, resin infiltrant and fissure sealant	x	
47	x		Enamel with different adhesive systems, etching using phosphoric acid	x	
48	x		Enamel and dentine without erosion suing soft drink and remineralisation	x	
49	x		Enamel exposed to soft drink, remineralised	x	
50	x		Enamel exposed to soft drink, remineralised	x	
51			Review		
52	x		Enamel with and without demineralisation using citric acid, remineralisation	x	
53			Dentine, review tooth structure	x	
54	x		Enamel and dentine with and without exposure to soft drink, remineralised	x	
55	x		Dentine demineralised with phosphoric acid and with and without remineralisation	x	

56	x		Dentine demineralised with EDTA and remineralised	x	
57	x		Carious teeth, deproteinization, infiltration		
58	x		Enamel with and without etching using phosphoric acid	x	
59	x		Enamel, scratch, washing, wear test	x	
60	x		Enamel exposed to HCl	x	
61	x		Enamel, scratches, review	x	
62	x		Enamel with and without exposition to Mg ions	x	
63	x		Enamel with non-cavitated lesion, with and without resin, etching using HCl		
64	x				
65	x		Enamel, erosion	x	
8	x		Dentine exposed to acetate buffer, remineralised	x	
66	x				
67	x		Enamel etched with orthophosphoric acid	x	
68			Dentine exposed to ethyle-nediaminetetraacetic acid, adhesive, brush		
69,70	x		Enamel before and after erosion using dietary acid		
71	x		Dentine demineralised using lactate or acetate buffer, remineralisation	x	
72	x		Carious tooth, dentine, with and without restoration	x	
73	x		Enamel with and without etching using phosphoric acid, remineralised	x	
74	x		Enamel, erosion using citric acid, and abrasion	x	
75,76	x		Enamel, interproximal reduction	x	

77	x		Enamel etched using citric acid, before and after scratch test, plucking test	x	
78	x		Root caries	x	
79	x		Enamel, erosion	x	
80	x		Teeth with and without exposition to liquid medicaments	x	
81	x		Enamel, impact and sliding wear experiment, microcracks		
82	x		Enamel with and without etching using phosphoric acid, remineralised	x	
83	x		Enamel demineralised using phosphoric acid		
84	x		Dentine	x	
85	x		Enamel with different polishing and grinding process, etched with phosphoric acid	x	
86	x		Enamel demineralised using phosphoric acid and remineralised	x	
87	x		Enamel exposed to soft drinks	x	
88	x		56		
89	x		Dentine with and without demineralisation using phosphate buffer, and remineralisation	x	
90	x		Enamel, indentation	x	
91	x		Teeth brushed, enamel, dentine, cementum	x	
92	x	x	Enamel etched with phosphoric acid	x	
93	x		Enamel before and after deproteinisation	x	
94	x		Enamel demineralised using at least acetic acid, and remineralised		
95			Review		
96	x		Dentine, fractured	x	
1	x		Enamel exposed to soft drinks	x	

97	x		Enamel with white spot lesion, normal enamel, dentine	x	
98	x		Enamel, predebonding, tungsten carbide bur, polishing	x	
99	x		Teeth with and without demineralisation using orthophosphoric acid, remineralised	x	
100	x		Enamel exposed to test foods	x	
101	x		Dentine demineralised using acetic acid, and remineralised, toothbrush, deionized water	x	
102	x		Teeth	x	
103	x		Tooth brushed, dentine, enamel, cementum	x	
104	x		Dentine treated with carboxymethyl chitosan or phosphoric acid	x	
105	x		Teeth from healthy and chronic renal failure, predialysis groups, whitening protocol, before and after bleaching	x	
106	x		Enamel with and without etching with phosphoric acid, composite resin, brush	x	
107	x		Enamel, dentine, laser irradiation	x	
108	x		Dentine, with and without dentinogenesis imperfect II	x	

(a)

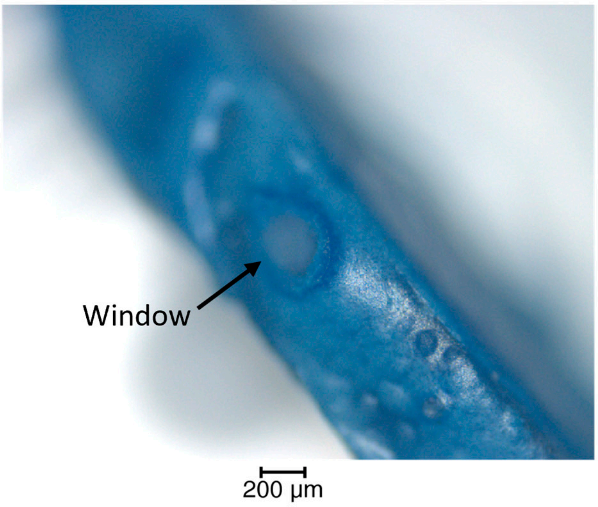

(b)

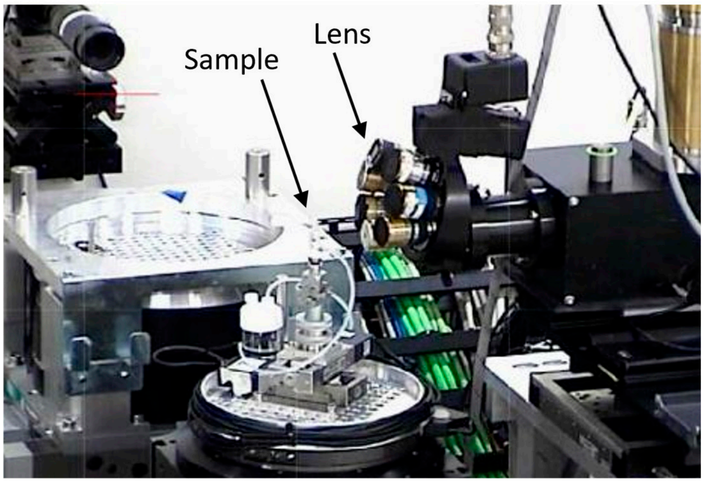

SI-Fig. S1. Setup of the tomography experiment. (a) Light microscopy image of the sample prior to the synchrotron experiment. (b) Setup of the synchrotron tomography experiment.

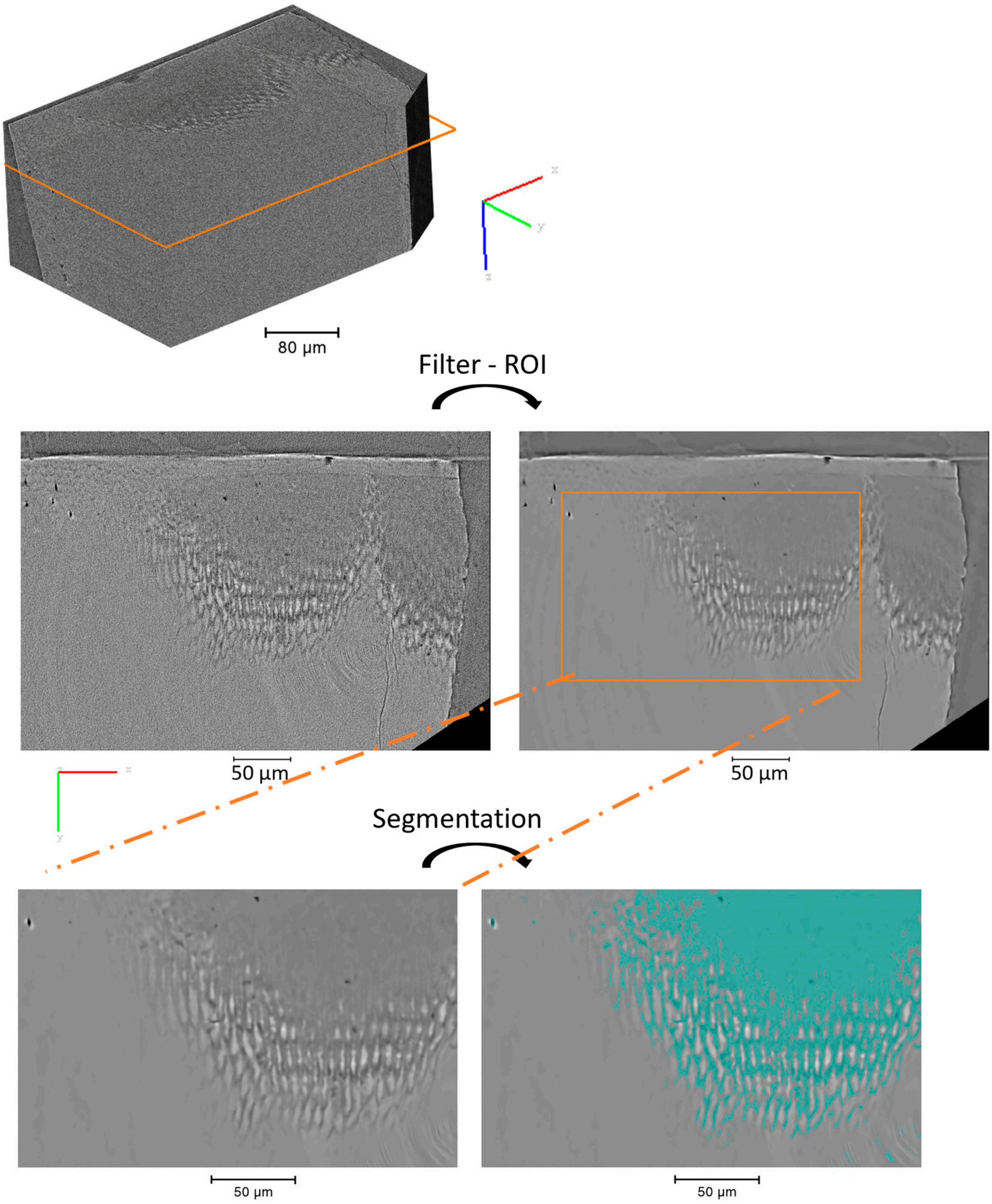

SI-Fig. S2. Details of the process of the segmentation of the lesion. Volume rendering of the raw data after alignment, and illustration of a slice before and after filtering with the detail of the region of interest (ROI). Overlap of the segmented region in blue with the slice of enamel.

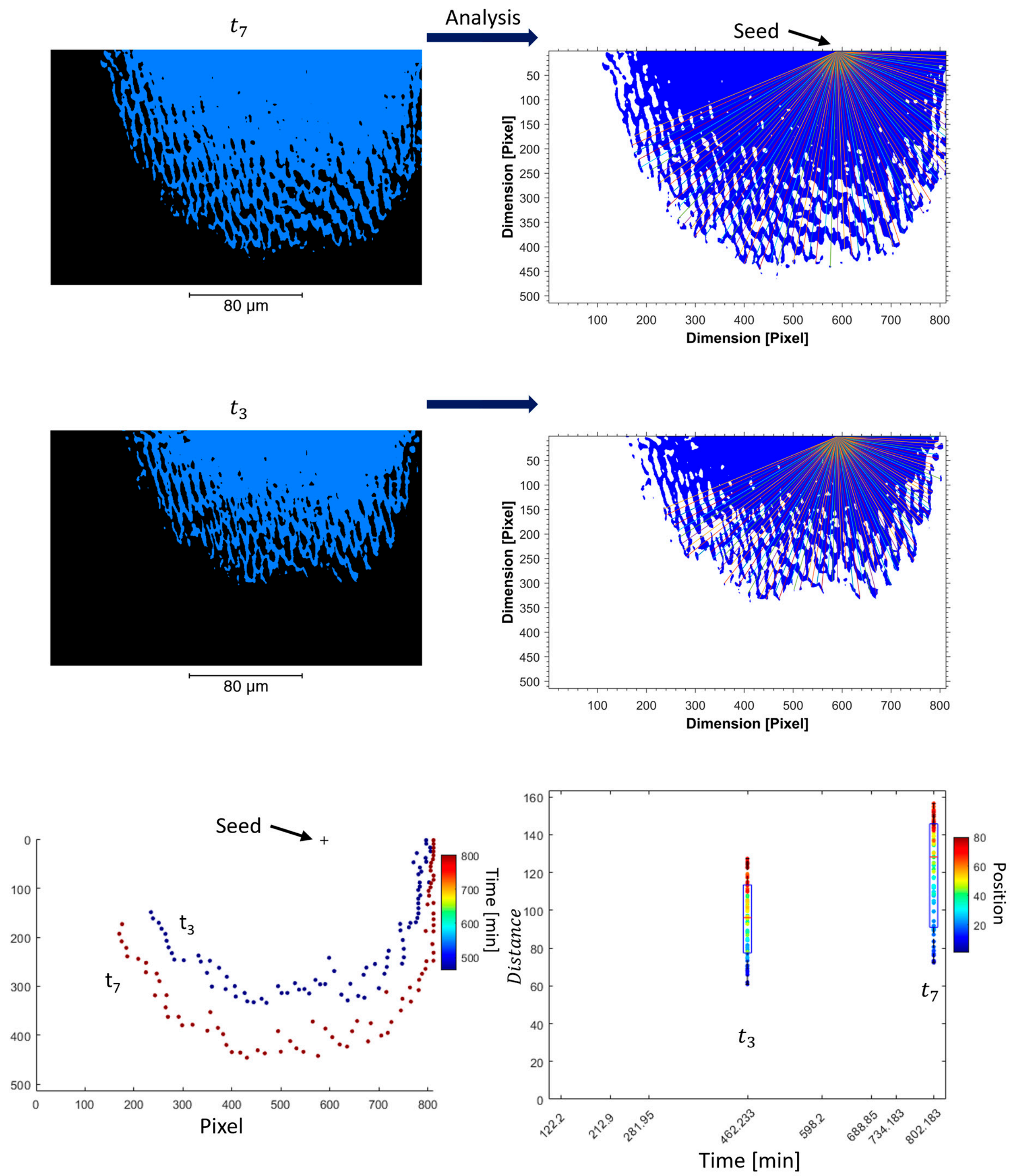

SI-Fig. S3. Process of the analysis of the distance. Illustration of the analysis of the ‘radius distance’ on one segmented virtual slice extracted from t_7 and t_3. Visualisation of the seed and the different lines based on the angles (referred to as position). A plot of the distance d_{t_i} for t_7 and t_3. The lines were done starting from 0 ° and ending at 157.5 °, in total 80 lines equally spaced (~ 1.9937 °).

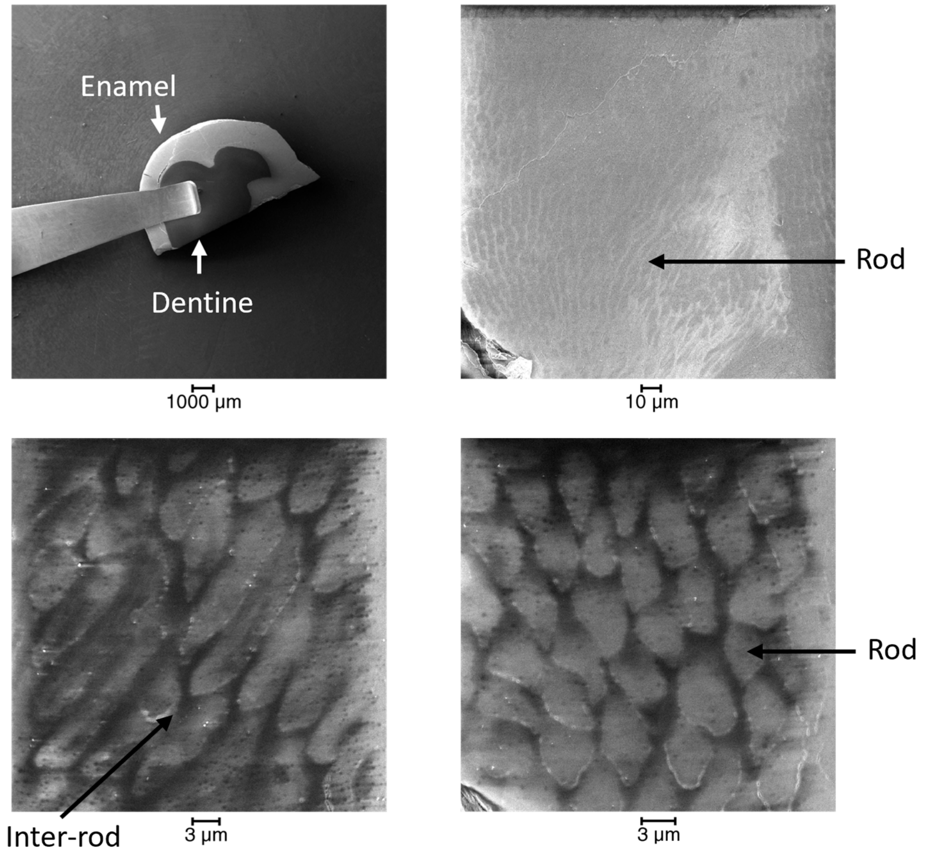

SI-Fig. S4. Scanning electron microscopy (SEM) of the slice before the AFM analysis. SEM analysis with the secondary electron images of the slice prior to the SEM and the observation of the enamel structure.

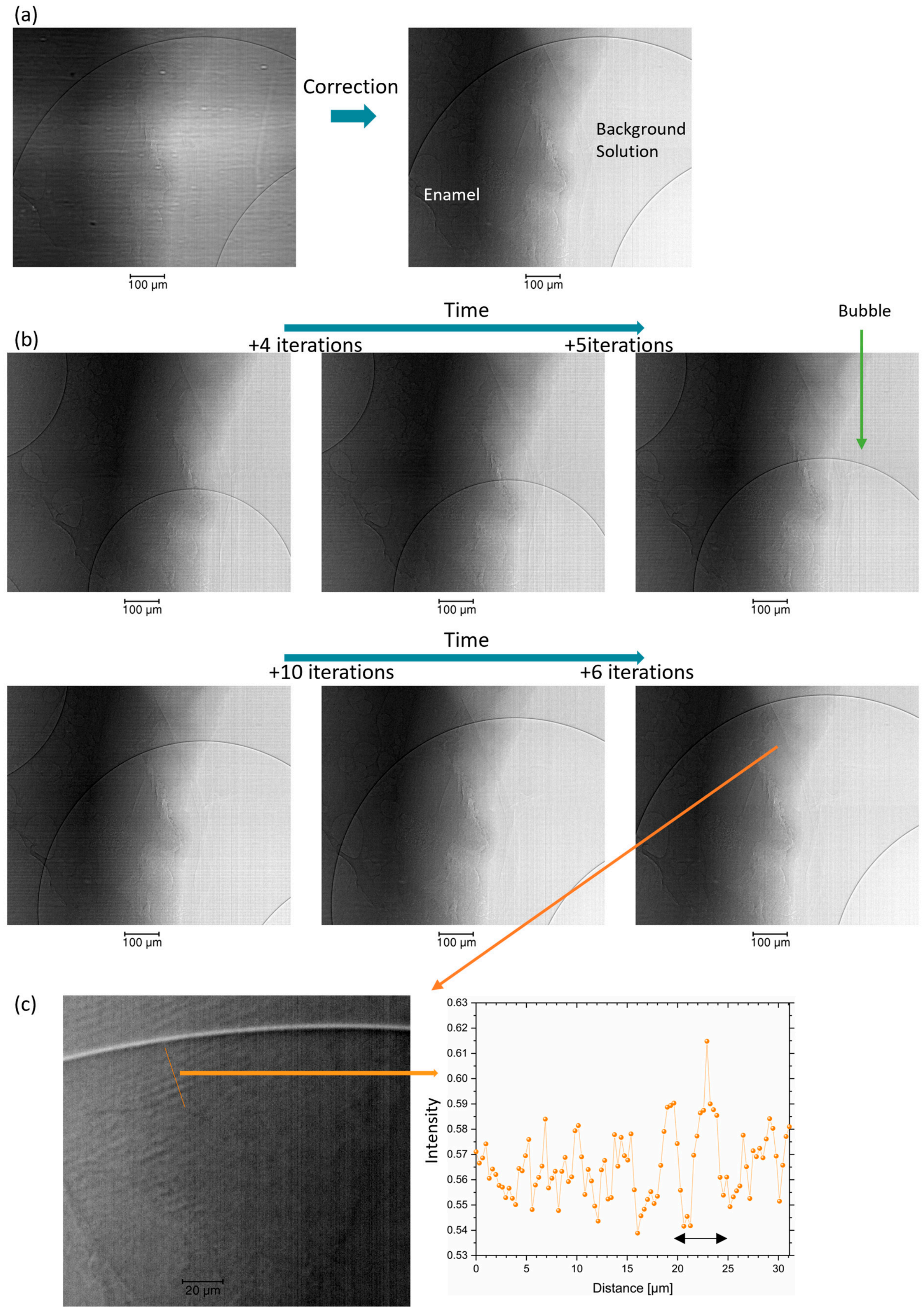

SI-Fig. S5. Projections of the tomography data with and without correction. (a) One projection image of the last data point before and after flat and dark field correction. (b) Time-lapse of the corrected

projections images. (c) Zoom in on the last data point of the projection with representation using an invert colour map, line profile analysis, and the highlight of the scale for the distance.

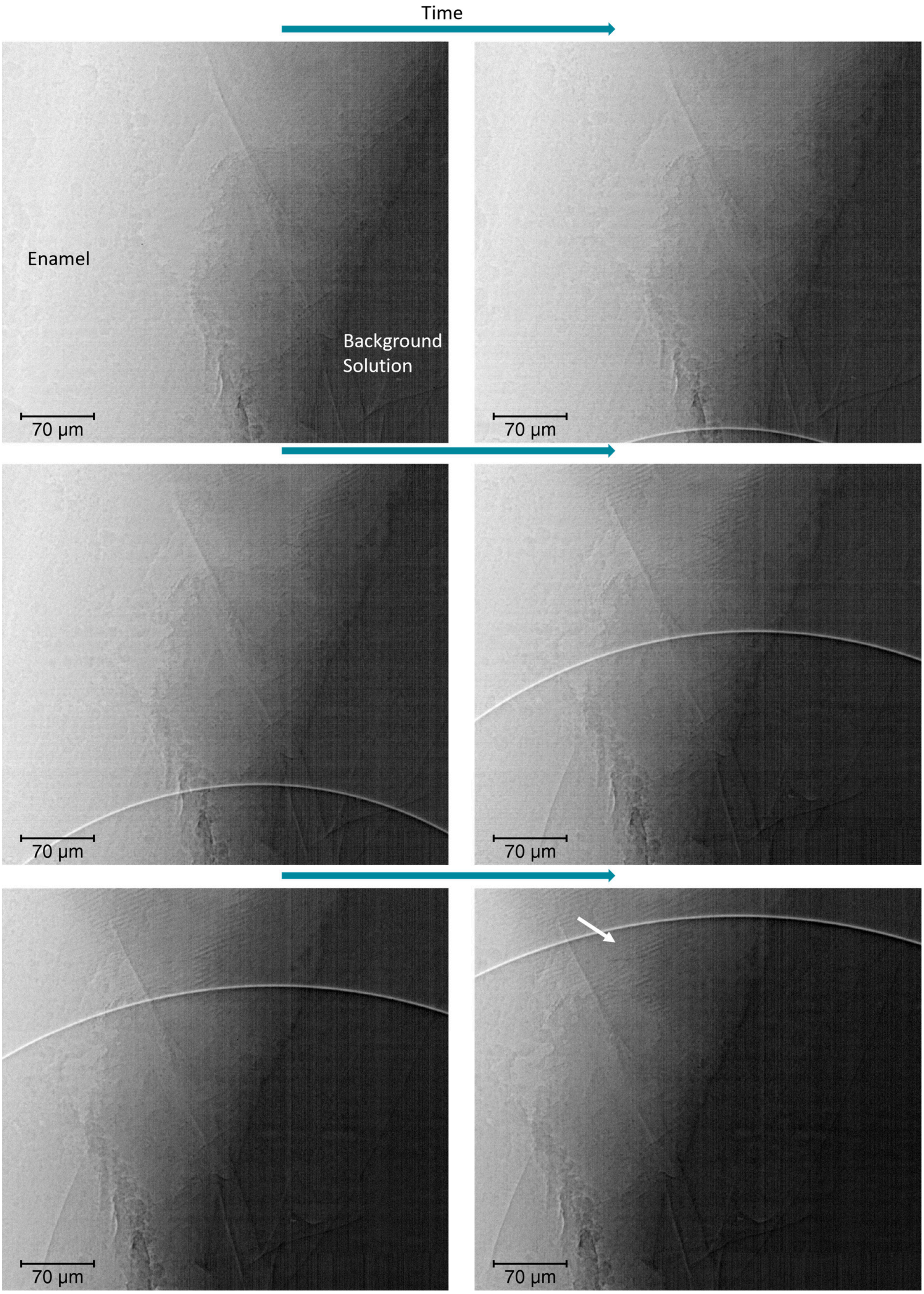

SI-Fig. S6. Zoom in on the projections images. Time-lapse of the projections from several datapoints, illustrated with an invert colour map. Structure highlighted with an arrow.

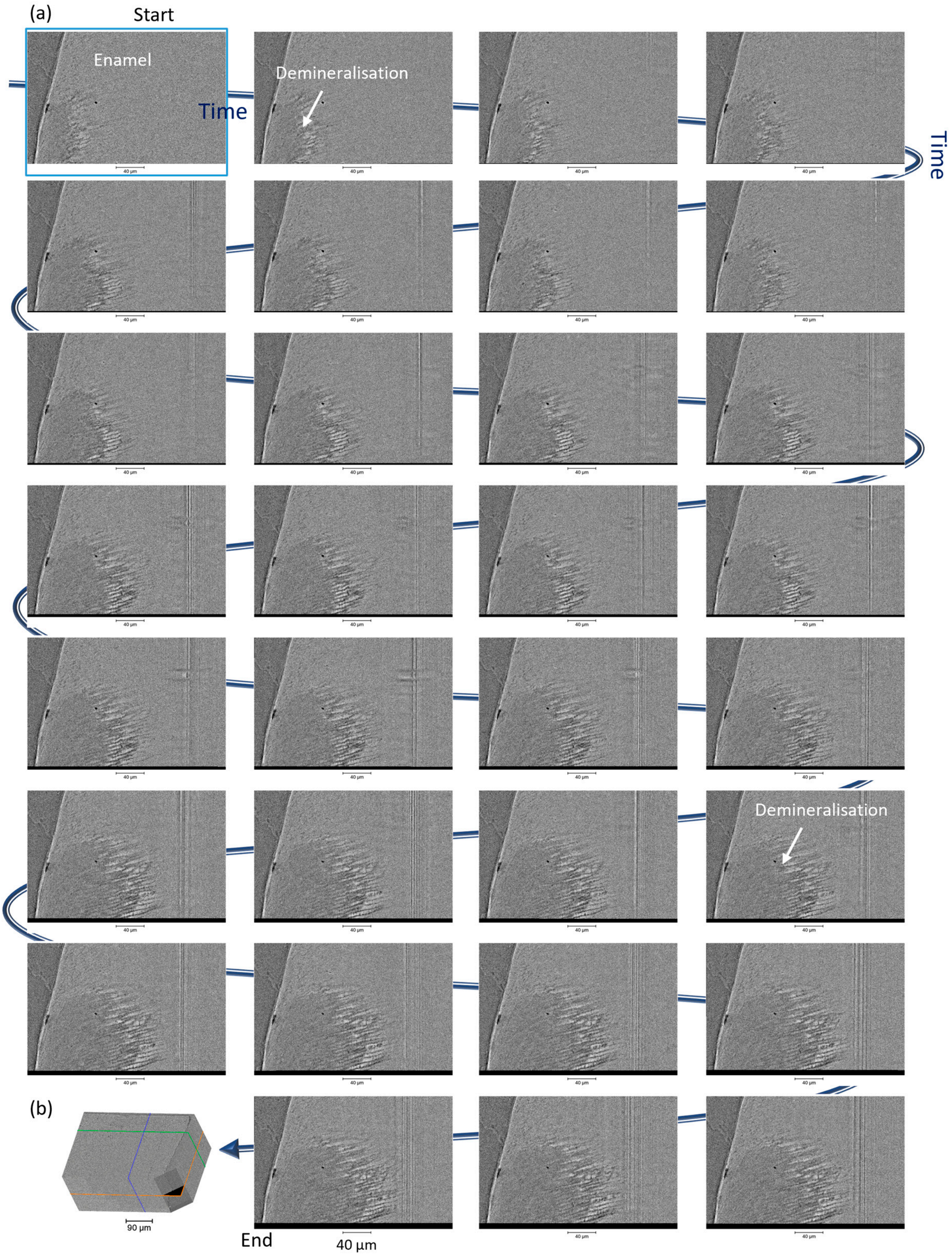

SI-Fig. S7. Time-lapse of slice from another orientation of each dataset. Virtual slices, from the first time point t_0 to the last time point t_{end} (scale bar 50 µm). Location of the slice was shown in Figure 2.

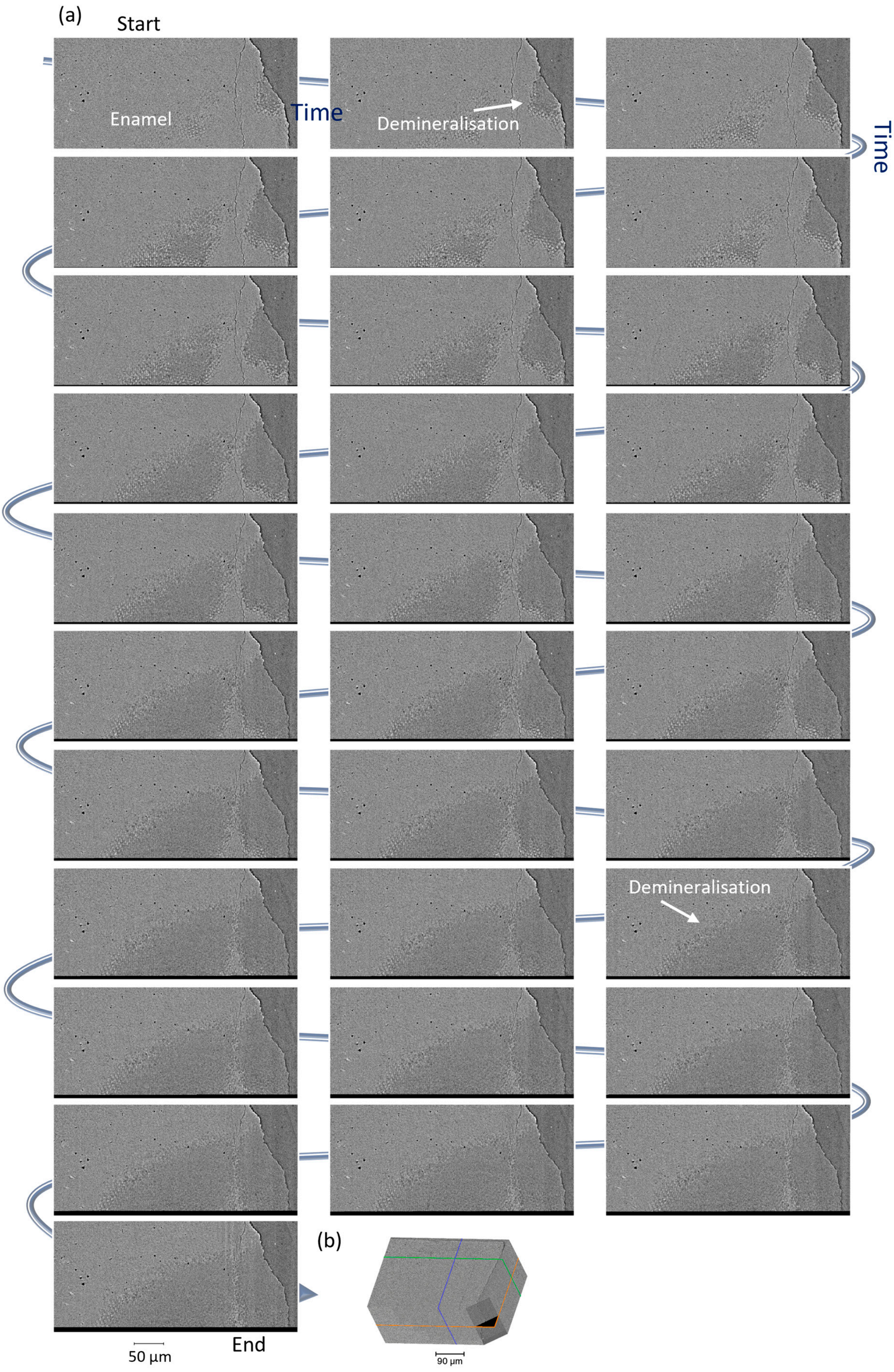
(a)
Start
Enamel
Time
Demineralisation
Time
Demineralisation
50 µm
End
(b)
90 µm

SI-Fig. S8. Time-lapse of slice from another orientation of each dataset. Virtual slices, from the first time point t_0 to the last time point t_{end} (scale bar 50 µm). Location of the slice was shown in Figure 2.

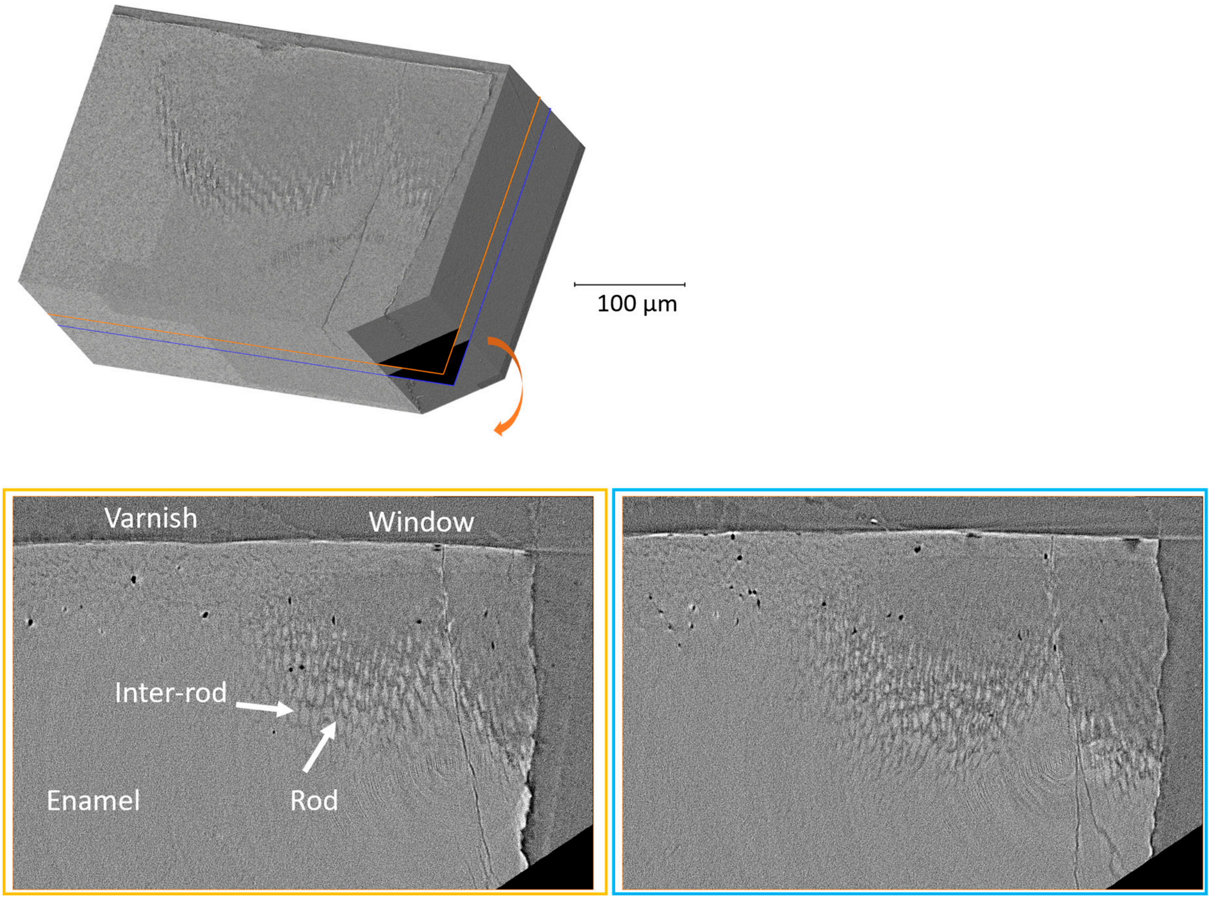

SI-Fig. S9. Analysis of the dataset from the last time point. 3D rendering of the dataset with the position of two virtual slices. Virtual slices from the last time point and details of the features seen after reconstruction, rods and inter-rods.

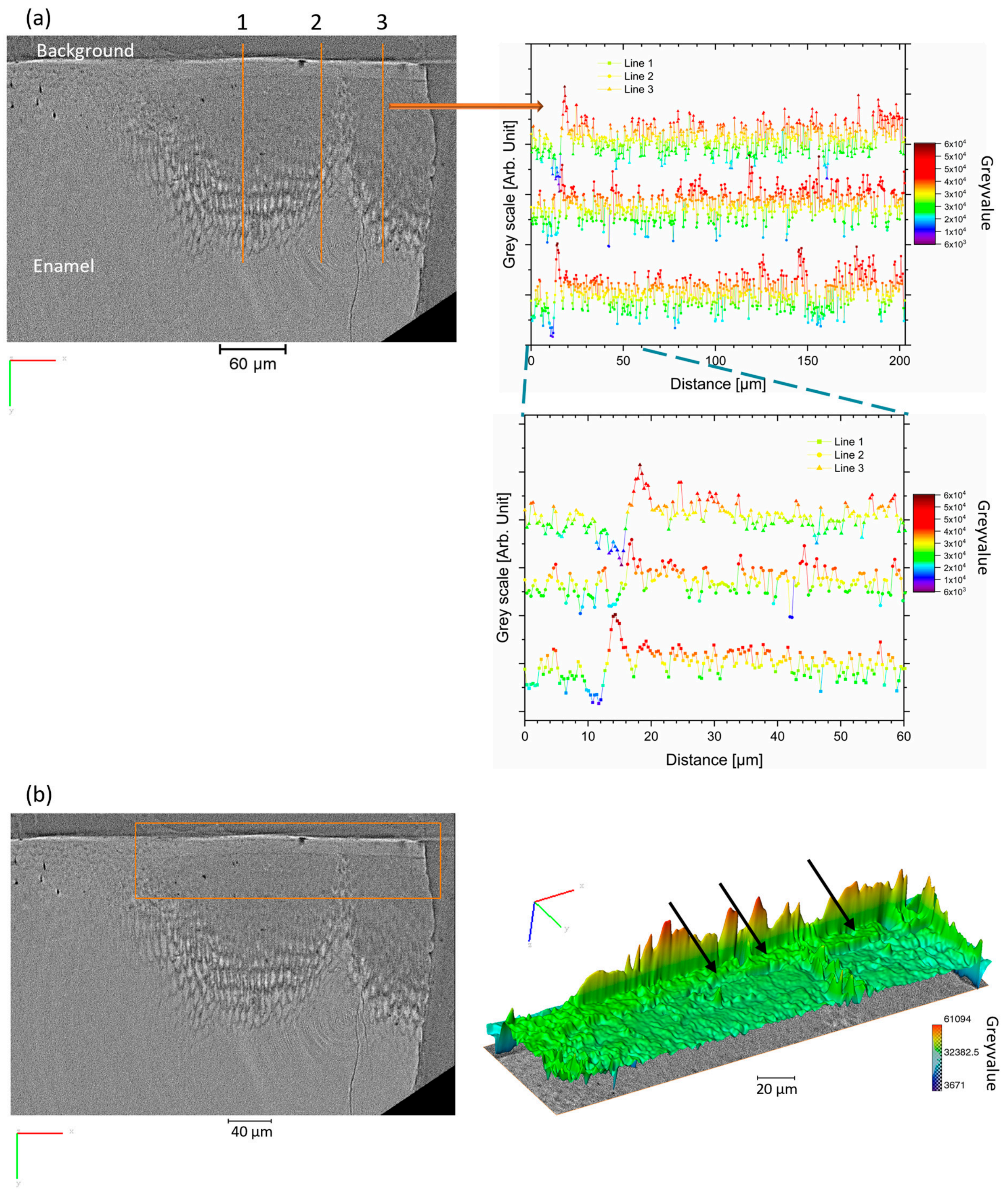

SI-Fig. S10. Surface enamel details. Line scan taken along the xy axis of the volume rendering of the last data point, and this shows the variation of grey scale from outside the enamel to the core of the enamel. Three positions are taken for illustration. (b) Rendering of the grey scale in a region of interest overlapped with a virtual slice.

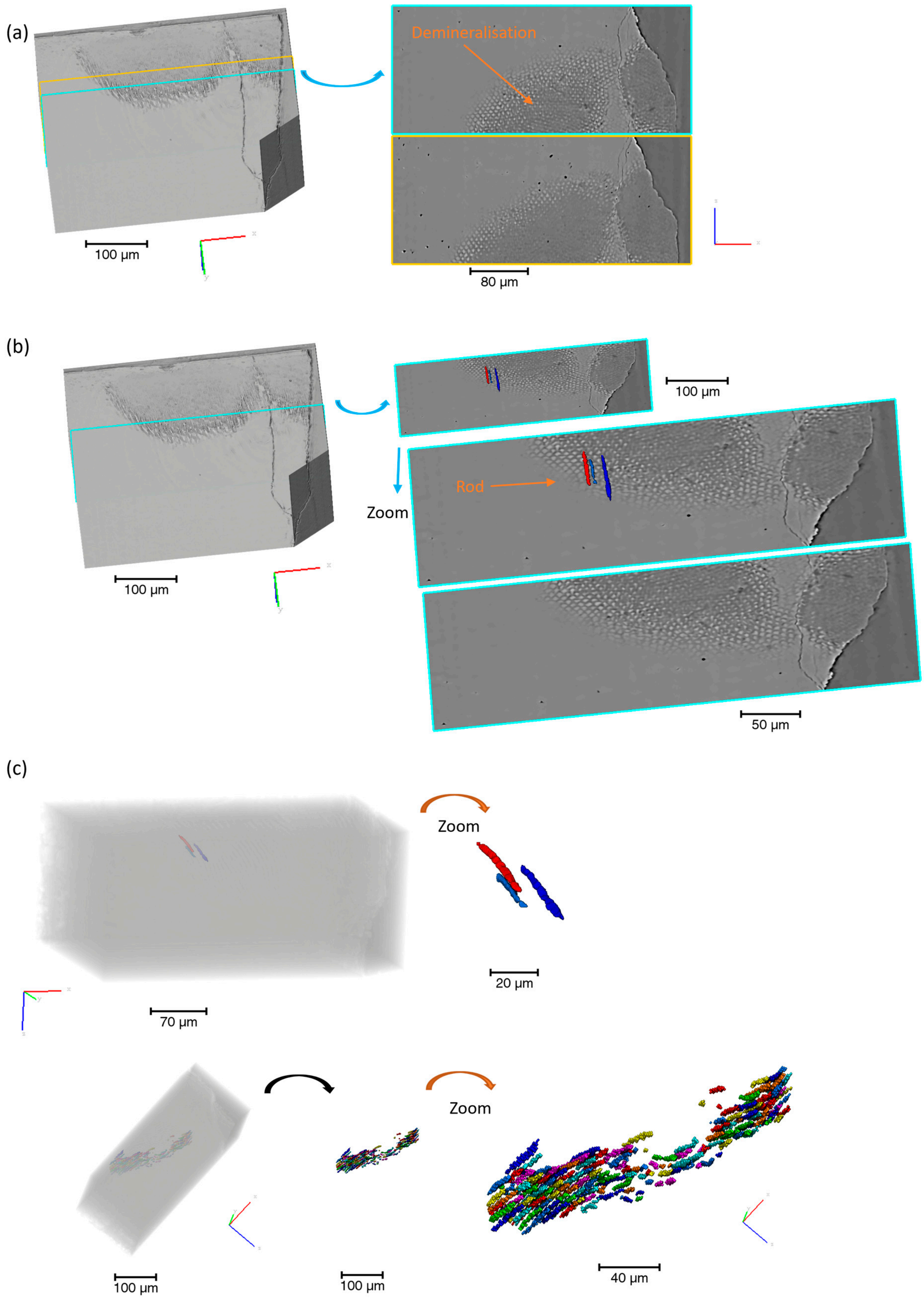

SI-Fig. S11. Analysis of the non-demineralised region and illustration of the rod structure. (a) 3D rendering of enamel with the highlight of two virtual slices (1280 × 871 × 550 pixels). (b) 3D rendering

of the enamel and rods with the position in the sample (datasets after median and non-local mean filter using Avizo). (c) Zoom in the rods and 3D rendering of more rods. The segmentation of the rods was carried out with Avizo using the segmentation editor to illustrate the locations.

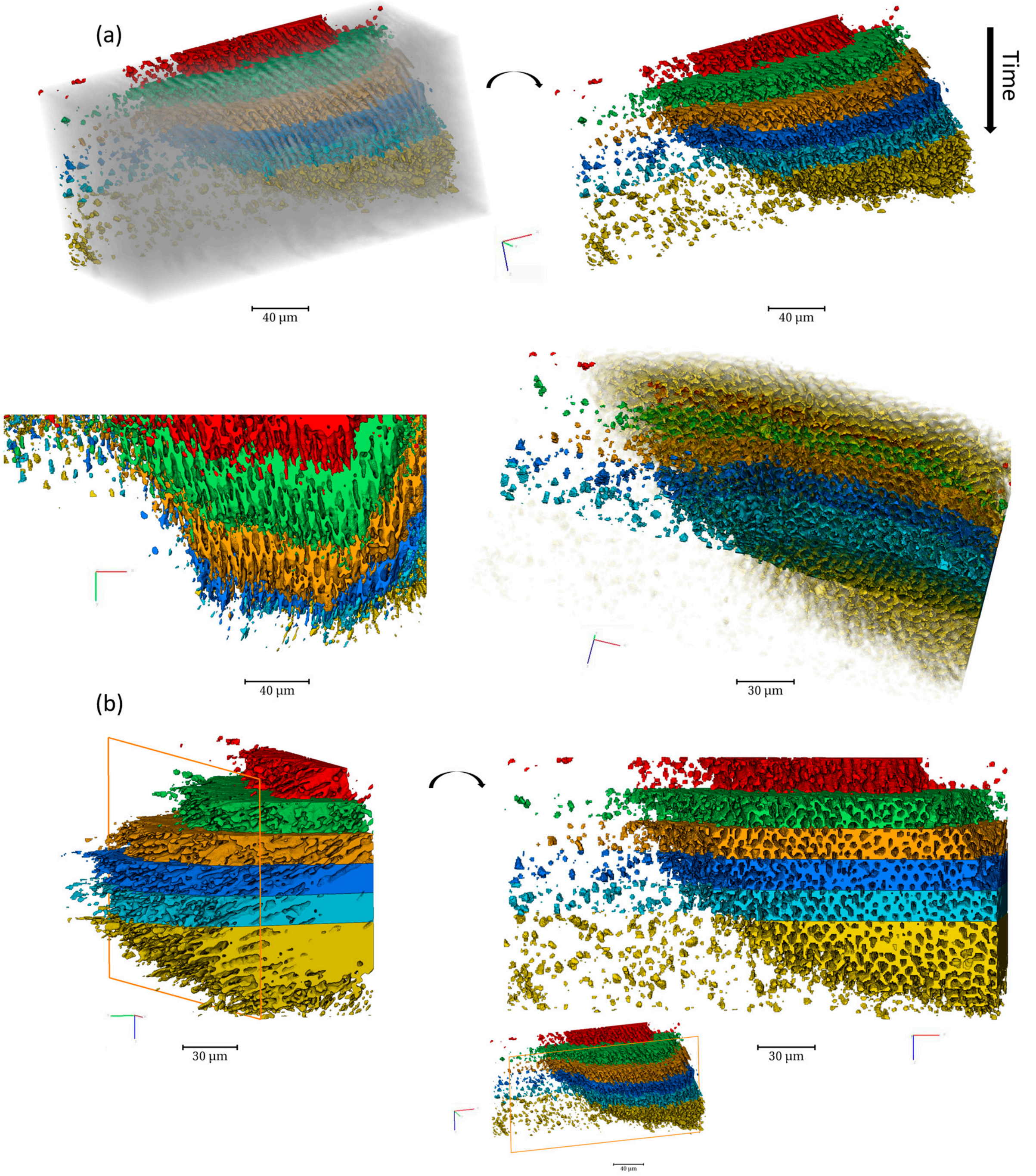

SI-Fig. S12. Volume rendering of several datasets described in Figure 3. (a) Several views of the superimposition of the segmented dataset at the different time points, with red colour being the start and yellow the last iteration in the datasets acquired. (b) A virtual cut of the volumes along the plane was shown in orange.

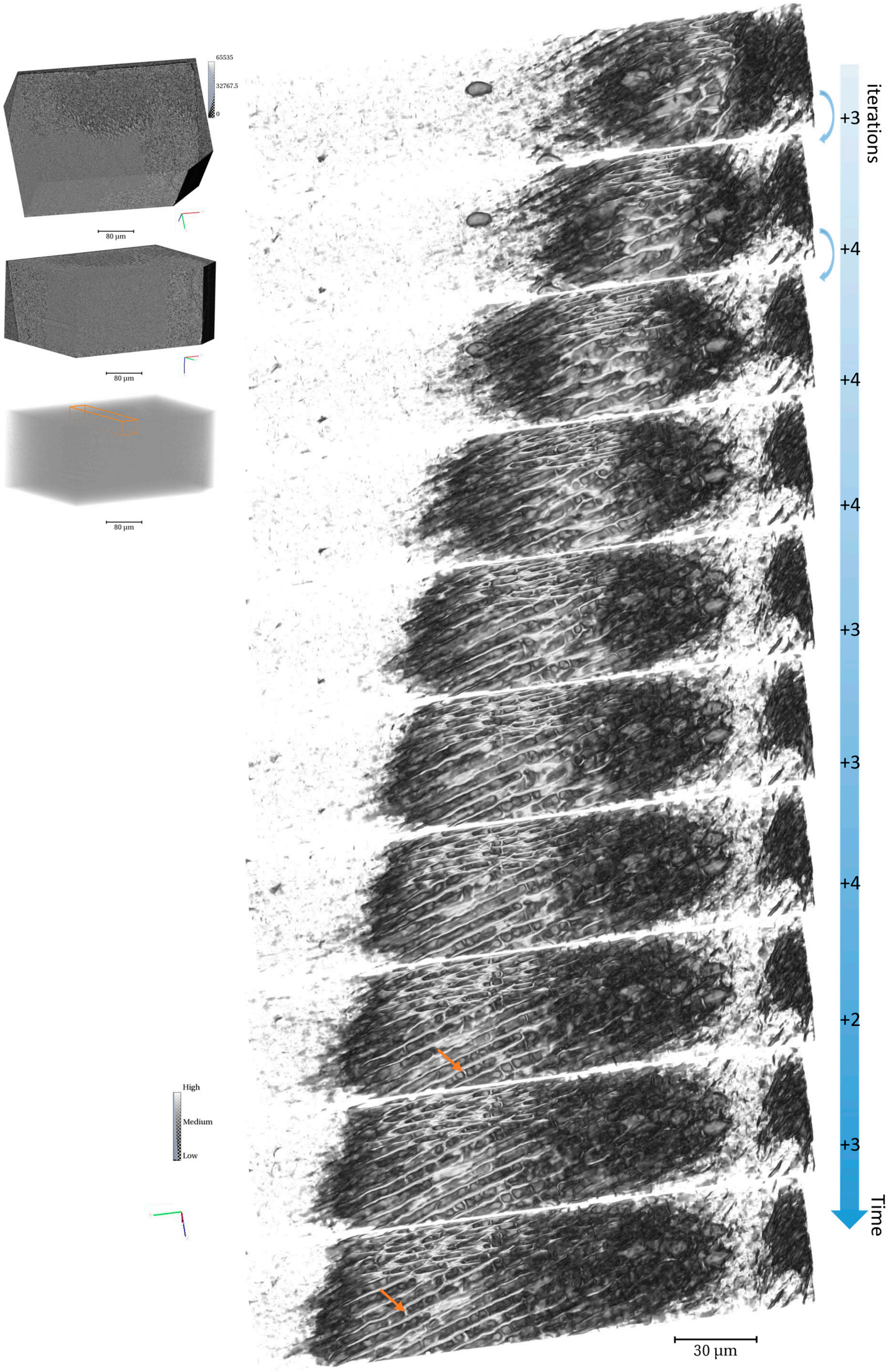
65535
32767.5
0
80 µm
80 µm
80 µm
High
Medium
Low
+3
+4
+4
+4
+3
+3
+4
+2
+3
iterations
Time
30 µm

SI-Fig. S13. Analysis of the demineralised regions with time. Volume rendering from one time point and the highlight of a region of interest in orange. Progression of the demineralisation with time (iterations shown corresponding to the tomograms, '+3' referred to as 3 tomograms passed, last image being the last iteration) showing the pathway of the acid and modification of the enamel structure, with rods, inter-rods and striations (highlighted with orange arrows). The images of the progression were processed using a membrane enhancement filter in Avizo on the non-filtered dataset.

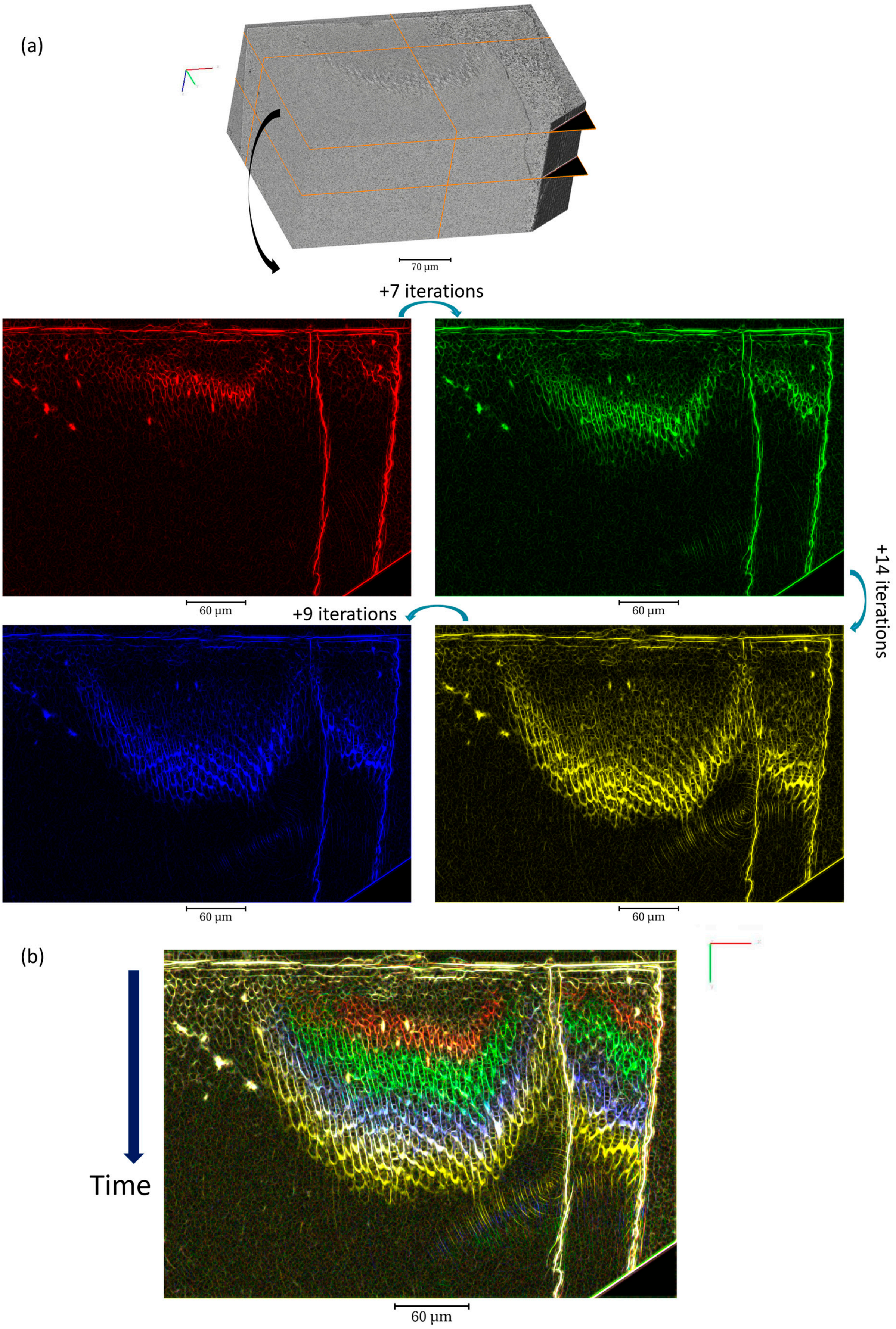
(a)
70 µm
+7 iterations
60 µm
+9 iterations
60 µm
+14 iterations
60 µm
60 µm
(b)
Time
60 µm

SI-Fig. S14. Progression of the demineralisation with time. (a) 3D rendering of the enamel with the highlight of the position of virtual slices along different axes. Virtual slices at four time points from Figure 3, showed the progression of the demineralised region along the rods and inter-rods (yellow image, the last iteration, the iterations corresponded to the tomograms, '+7' referred to as 7 tomograms passed). These were filtered images similar to SI-Fig. S13. (b) Superimposition of the slices.

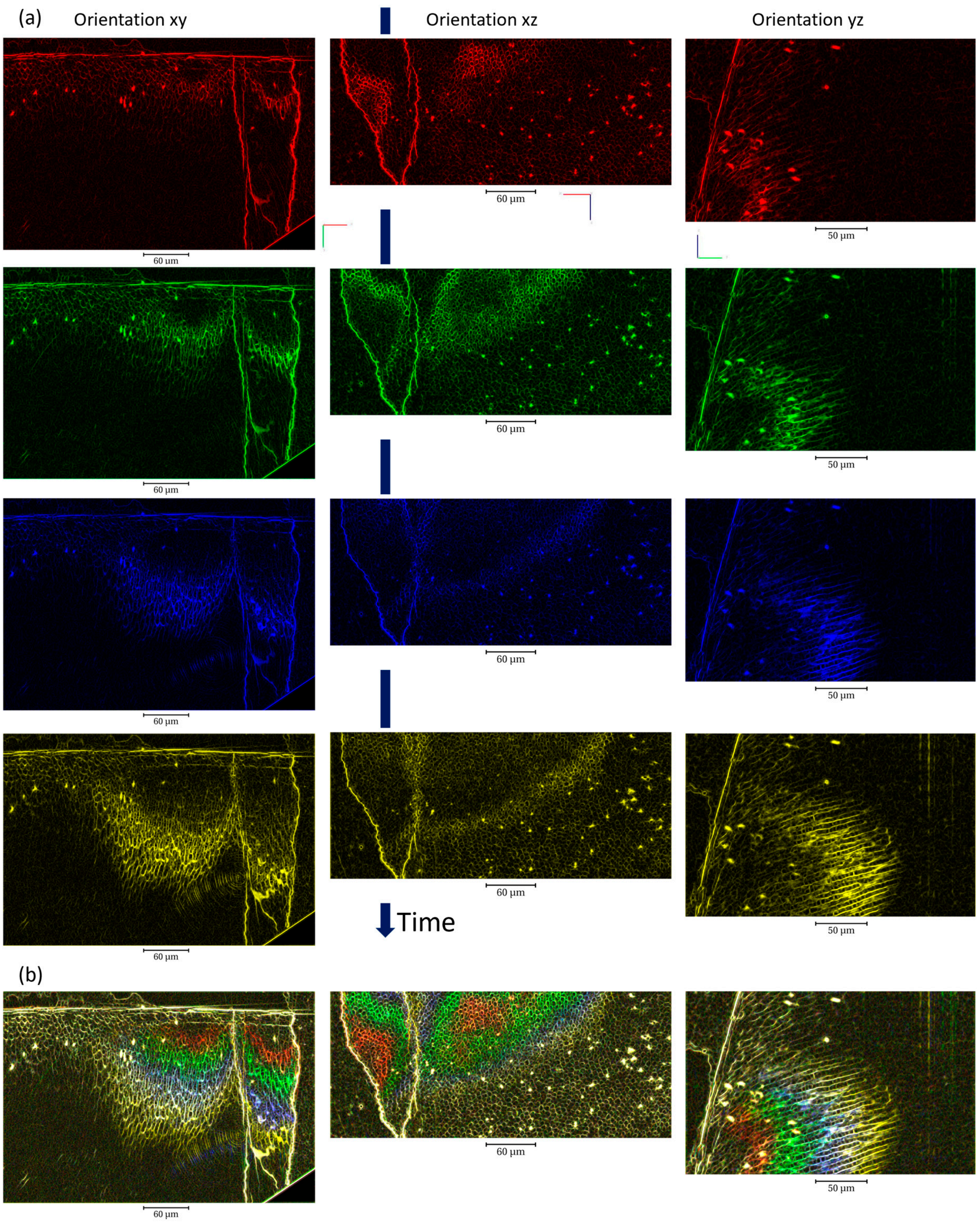

SI-Fig. S15. Progression of the demineralisation with time along different axes. (a) Virtual slices at four time points from Figure 3, showed the progression of the demineralised region along the rods and inter-rod. The positions of the slices were detailed in SI-Fig. S14a. These were filtered images similar to SI-Fig. S13. (b) Superimposition of the slices. The colour code was detailed in SI-Fig. S14 with the details of the iterations illustrated.

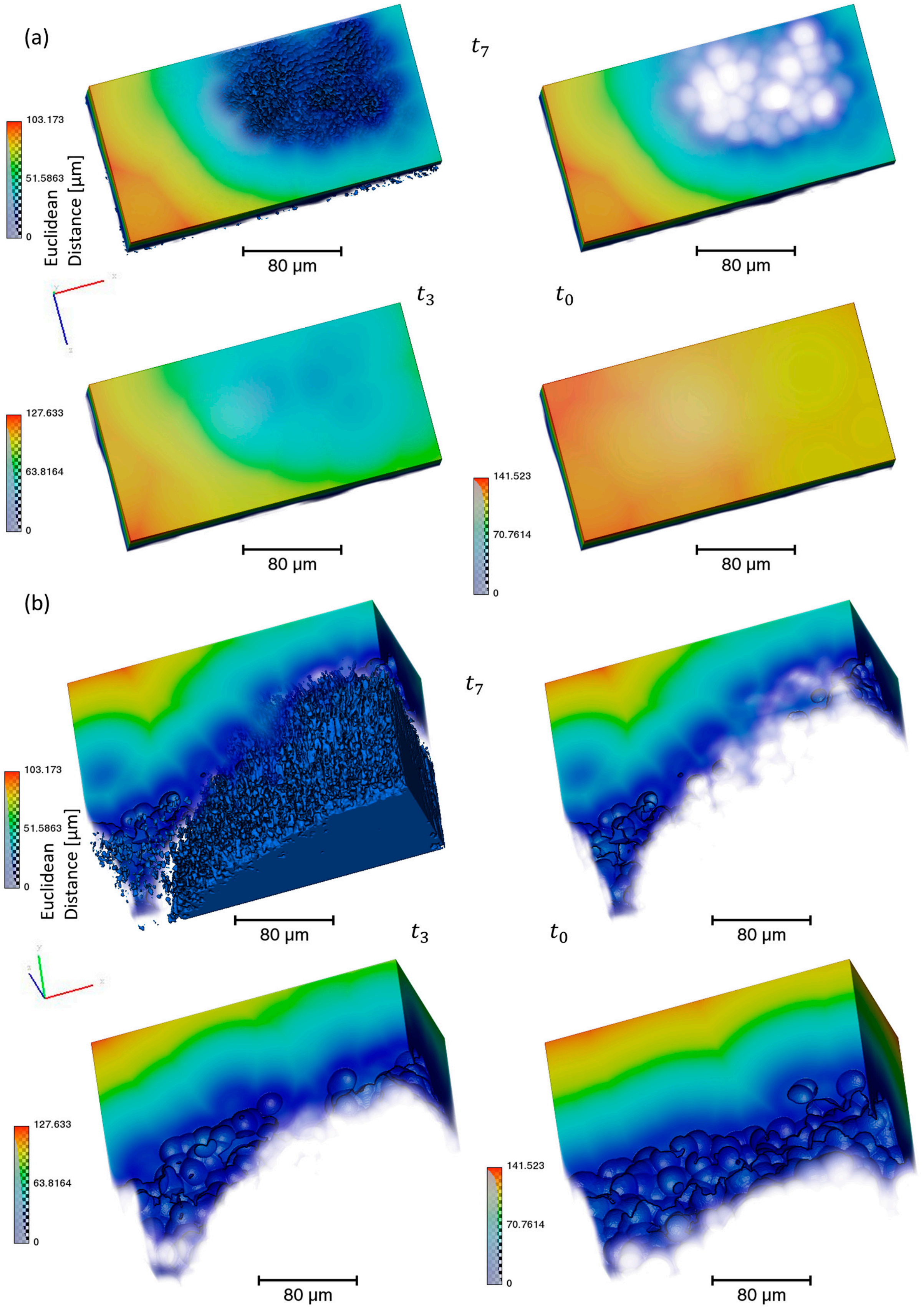

SI-Fig. S16. Distance computed from several time points of the remaining region. (a) and (b) two orientations of the distance computed from t_7, t_0, and t_3, with an overlap of the segmented region for t_7.

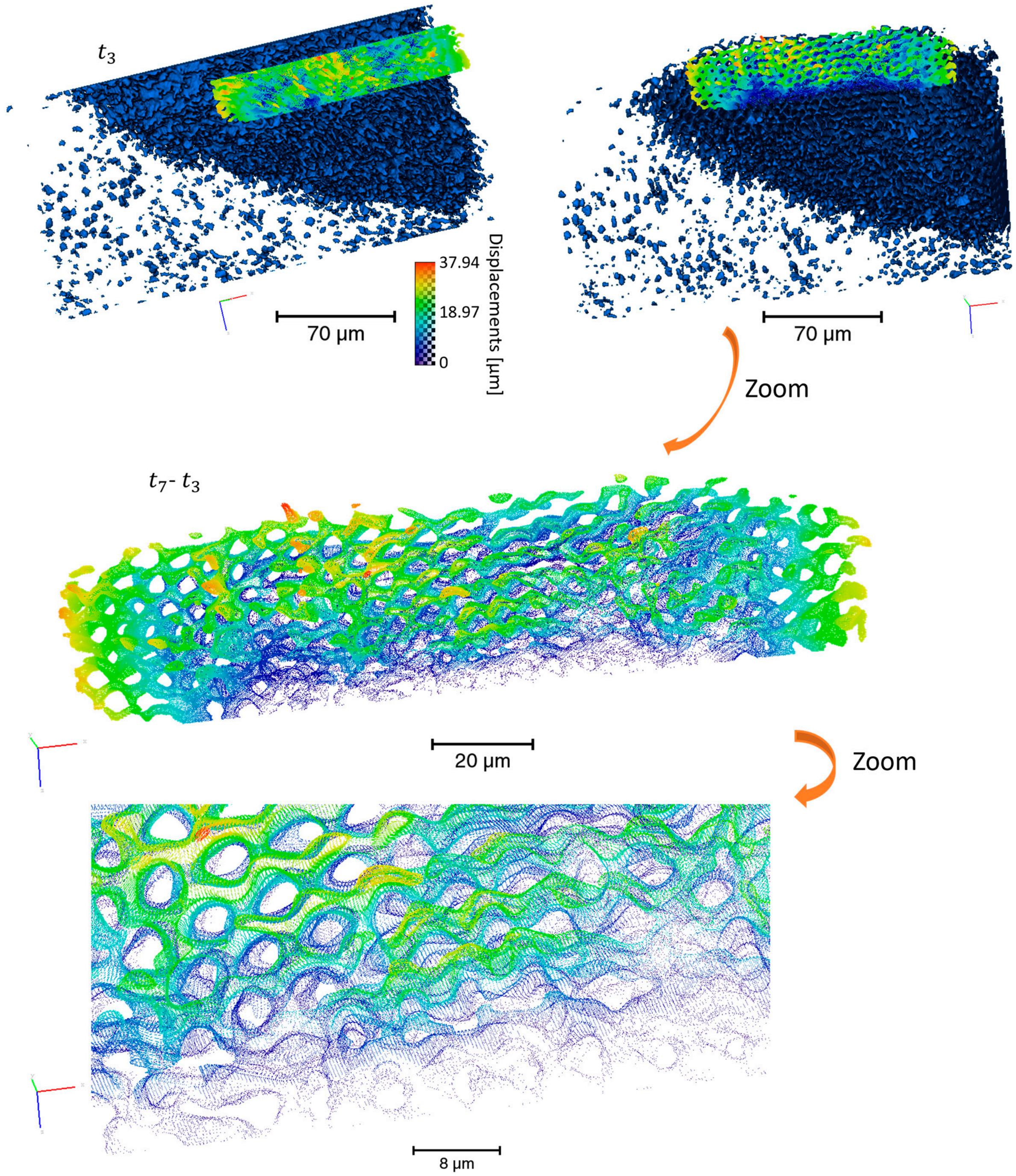

SI-Fig. S17. Illustration of the anisotropy of the progression of the lesion from t_3, to t_7. Volume rendering of the dataset at t_3 (812 × 514 × 400 pixels) with the progression of the lesion between t_7 and t_3 from a sub-region (506 × 208 × 88 pixels), and zoom-in in the 3D region of the progression with distance information in µm in 3D. This was carried out using the module Surface distance in Avizo (surface from t_7 to t_3) from the surfaces of the two time points over a region of interest followed by the visualisation of the surface vectors of the displacements.

Movies

Movie S1. Time-lapse of the progression of the demineralisation with time which is shown using the virtual slices described in Figure 2.

Movie S2. Volume rendering of the volumes of the segmented datasets described in Figure 3 with virtual cut showing the progression in the sample.

Movie S3. Virtual slice of the segmented datasets described in Figure 3, last iteration t_7 (in yellow) and t_4 (in blue). Iterations from the description in Figure 4.

Movie S4. Volume rendering of t_7. Volume rendering of the segmented volume of t_7 and virtual cut to show the progression through the volume. Additional details in Figures 3,4.

References

1 Li, P., Oh, C., Kim, H., Chen-Glasser, M., Park, G., Jetybayeva, A., Yeom, J., Kim, H., Ryu, J. & Hong, S. Nanoscale effects of beverages on enamel surface of human teeth: an atomic force microscopy study. *Journal of the Mechanical Behavior of Biomedical Materials* **110**, 103930, doi:https://doi.org/10.1016/j.jmbbm.2020.103930 (2020).

2 Ge, J., Cui, F. Z., Wang, X. M. & Feng, H. L. Property variations in the prism and the organic sheath within enamel by nanoindentation. *Biomaterials* **26**, 3333-3339, doi:10.1016/j.biomaterials.2004.07.059 (2005).

3 Burwell, A. K., Thula-Mata, T., Gower, L. B., Habeliz, S., Kurylo, M., Ho, S. P., Chien, Y.-C., Cheng, J., Cheng, N. F., Gansky, S. A., et al. Functional remineralization of dentin lesions using polymer-induced liquid-precursor process. *PLoS ONE* **7**, e38852, doi:10.1371/journal.pone.0038852 (2012).

4 Kim, S. A., Smith, S., Beauchamp, C., Song, Y., Chiang, M., Giuseppetti, A., Frukhtbeyn, S., Shaffer, I., Wilhide, J., Routkevitch, D., et al. Cariogenic potential of sweet flavors in electronic-cigarette liquids. *PLoS ONE* **13**, e0203717, doi:10.1371/journal.pone.0203717 (2018).

5 De Yoreo, J. J., Chung, S. & Friddle, R. W. In situ atomic force microscopy as a tool for investigating interactions and assembly dynamics in biomolecular and biomineral systems. *Advanced Functional Materials* **23**, 2525-2538, doi:https://doi.org/10.1002/adfm.201203424 (2013).

6 Bai, Y., Yu, Z., Ackerman, L., Zhang, Y., Bonde, J., Li, W., Cheng, Y. & Habelitz, S. Protein nanoribbons template enamel mineralization. *Proceedings of the National Academy of Sciences* **117**, 19201-19208, doi:10.1073/pnas.2007838117 (2020).

7 Wallwork, M. L., Kirkham, J., Zhang, J., Smith, D. A., Brookes, S. J., Shore, R. C., Wood, S. R., Ryu, O. & Robinson, C. Binding of matrix proteins to developing enamel crystals: an atomic force microscopy study. *Langmuir* **17**, 2508-2513, doi:10.1021/la001281r (2001).

8 Chien, Y.-C., Tao, J., Saeki, K., Chin, A. F., Lau, J. L., Chen, C.-L., Zuckermann, R. N., Marshall, S. J., Marshall, G. W. & De Yoreo, J. J. Using biomimetic polymers in place of noncollagenous proteins to achieve functional remineralization of dentin tissues. *ACS Biomaterials Science & Engineering* **3**, 3469-3479, doi:10.1021/acsbiomaterials.7b00378 (2017).

9 Moore, S., Burrows, R., Kumar, D., Kloucek, M. B., Warren, A. D., Flewitt, P. E. J., Picco, L., Payton, O. D. & Martin, T. L. Observation of stress corrosion cracking using real-time in situ high-speed atomic force microscopy and correlative techniques. *npj Materials Degradation* **5**, 3, doi:10.1038/s41529-020-00149-y (2021).

10 Kinney, J. H., Balooch, M., Marshall, G. W. & Marshall, S. J. Atomic-force microscopic study of dimensional changes in human dentine during drying. *Archives of Oral Biology* **38**, 1003-1007, doi:https://doi.org/10.1016/0003-9969(93)90114-2 (1993).

11 Marshall Jr., G. W., Wu-Magidi, I. C., Watanabe, L. G., Inai, N., Balooch, M., Kinney, J. H. & Marshall, S. J. Effect of citric acid concentration on dentin demineralization, dehydration, and rehydration: atomic force microscopy study. *Journal of Biomedical Materials Research* **42**, 500-507, doi:10.1002/(sici)1097-4636(19981215)42:4<500::Aid-jbm4>3.0.Co;2-l (1998).

12 Silikas, N., Watts, D. C., England, K. E. & Jandt, K. D. Surface fine structure of treated dentine investigated with tapping mode atomic force microscopy (TMAFM). *Journal of dentistry* **27**, 137-144, doi:10.1016/s0300-5712(98)00032-3 (1999).

13 Silikas, N., Lennie, A. R., England, K. & Watts, D. C. AFM as a tool in dental research. *Microscopy and Analysis*, 19-21 (2001).

14 Habelitz, S., Marshall, S. J., Marshall, G. W., Jr. & Balooch, M. Mechanical properties of human dental enamel on the nanometre scale. *Archives of Oral Biology* **46**, 173-183, doi:10.1016/S0003-9969(00)00089-3 (2001).

15 Finke, M., Hughes, J. A., Parker, D. M. & Jandt, K. D. Mechanical properties of in situ demineralised human enamel measured by AFM nanoindentation. *Surface Science* **491**, 456-467, doi:10.1016/S0039-6028(01)01311-5 (2001).
16 Habelitz, S., Marshall, S. J., Marshall, G. W., Jr. & Balooch, M. The functional width of the dentino-enamel junction determined by AFM-based nanoscratching. *Journal of Structural Biology* **135**, 294-301, doi:10.1006/jsbi.2001.4409 (2001).
17 Barbour, M. E., Parker, D. M., Allen, G. C. & Jandt, K. D. Enamel dissolution in citric acid as a function of calcium and phosphate concentrations and degree of saturation with respect to hydroxyapatite. *European Journal of Oral Sciences* **111**, 428-433, doi:10.1034/j.1600-0722.2003.00059.x (2003).
18 Lippert, F., Parker, D. M. & Jandt, K. D. In vitro demineralization/remineralization cycles at human tooth enamel surfaces investigated by AFM and nanoindentation. *Journal of Colloid and Interface Science* **280**, 442-448, doi:10.1016/j.jcis.2004.08.016 (2004).
19 Lippert, F., Parker, D. M. & Jandt, K. D. In situ remineralisation of surface softened human enamel studied with AFM nanoindentation. *Surface Science* **553**, 105-114, doi:https://doi.org/10.1016/j.susc.2004.01.040 (2004).
20 Robinson, C., Connell, S., Kirkham, J., Shore, R. & Smith, A. Dental enamel—a biological ceramic: regular substructures in enamel hydroxyapatite crystals revealed by atomic force microscopy. *Journal of Materials Chemistry* **14**, 2242-2248, doi:10.1039/B401154F (2004).
21 Batina, N., Renugopalakrishnan, V., Casillas Lavín, P. N., Guerrero, J. C. H., Morales, M., Garduño-Juárez, R. & Lakka, S. L. Ultrastructure of dental enamel afflicted with hypoplasia: an atomic force microscopic study. *Calcified Tissue International* **74**, 294-301, doi:10.1007/s00223-002-1045-2 (2004).
22 Watari, F. *In situ* quantitative analysis of etching process of human teeth by atomic force microscopy. *Journal of Electron Microscopy* **54**, 299-308, doi:10.1093/jmicro/dfi056 (2005).
23 Porter, A. E., Nalla, R. K., Minor, A., Jinschek, J. R., Kisielowski, C., Radmilovic, V., Kinney, J. H., Tomsia, A. P. & Ritchie, R. O. A transmission electron microscopy study of mineralization in age-induced transparent dentin. *Biomaterials* **26**, 7650-7660, doi:https://doi.org/10.1016/j.biomaterials.2005.05.059 (2005).
24 Wang, L., Tang, R., Bonstein, T., Orme, C. A., Bush, P. J. & Nancollas, G. H. A new model for nanoscale enamel dissolution. *The Journal of Physical Chemistry B* **109**, 999-1005, doi:10.1021/jp046451d (2005).
25 Chon, Y.-E., Jung, I.-Y., Roh, B.-D. & Lee, C.-Y. The change of the configuration of hydroxyapatite crystals in enamel by changes of pH and degree of saturation of lactic acid buffer solution. *Journal of Korean Academy of Conservative Dentistry* **32**, 498-513 (2007).
26 Uskoković, V., Kim, M. K., Li, W. & Habelitz, S. Enzymatic processing of amelogenin during continuous crystallization of apatite. *Journal of Materials Research* **23**, 3184-3195, doi:10.1557/JMR.2008.0387 (2008).
27 Vitkov, L., Kastner, M., Kienberger, F., Hinterdorfer, P., Schilcher, K., Grunert, I., Dumfahrt, H. & Krautgartner, W. D. Correlations between AFM and SEM imaging of acid-etched tooth enamel. *Ultrastructural Pathology* **32**, 1-4, doi:10.1080/01913120701808065 (2008).
28 Machado, C., Lacefield, W. & Catledge, A. Human enamel nanohardness, elastic modulus and surface integrity after beverage contact. *Brazilian Dental Journal* **19**, 68-72 (2008).
29 Keinan, D., Radko, A., Smith, P. & Zilberman, U. Acid resistance of the enamel in primary second molars from children with down syndrome and cerebral palsy. *The Open Dentistry Journal* **3**, 132-136, doi:10.2174/1874210600903010132 (2009).
30 Cheng, Z.-J., Wang, X.-M., Cui, F.-Z., Ge, J. & Yan, J.-X. The enamel softening and loss during early erosion studied by AFM, SEM and nanoindentation. *Biomedical Materials* **4**, 015020, doi:10.1088/1748-6041/4/1/015020 (2009).
31 Loyola-Rodriguez, J. P., Zavala-Alonso, V., Reyes-Vela, E., Patiño-Marin, N., Ruiz, F. & Anusavice, K. J. Atomic force microscopy observation of the enamel roughness and depth

profile after phosphoric acid etching. *Journal of Electron Microscopy* **59**, 119-125, doi:10.1093/jmicro/dfp042 (2009).
32 Pedreira de Freitas, A. C., Espejo, L. C., Botta, S. B., Teixeira, F. d. S., Luz, M. A. A. C., Garone-Netto, N., Matos, A. B. & Salvadori, M. C. B. d. S. AFM analysis of bleaching effects on dental enamel microtopography. *Applied Surface Science* **256**, 2915-2919, doi:https://doi.org/10.1016/j.apsusc.2009.11.050 (2010).
33 Gracia, L. H., Rees, G. D., Brown, A. & Fowler, C. E. An in vitro evaluation of a novel high fluoride daily mouthrinse using a combination of microindentation, 3D profilometry and DSIMS. *Journal of Dentistry* **38**, S12-S20, doi:https://doi.org/10.1016/S0300-5712(11)70004-5 (2010).
34 Mahmoud, S. H., Elembaby Ael, S., Zaher, A. R., Grawish Mel, A., Elsabaa, H. M., El-Negoly, S. A. & Sobh, M. A. Effect of 16% carbamide peroxide bleaching gel on enamel and dentin surface micromorphology and roughness of uremic patients: an atomic force microscopic study. *European Journal of Dentistry* **4**, 175-182 (2010).
35 Parkinson, C. R., Shahzad, A. & Rees, G. D. Initial stages of enamel erosion: an *in situ* atomic force microscopy study. *Journal of Structural Biology* **171**, 298-302, doi:https://doi.org/10.1016/j.jsb.2010.04.011 (2010).
36 Choi, S., Rhee, Y., Park, J.-H., Lee, G.-J., Kim, K.-S., Park, J.-H., Park, Y.-G. & Park, H.-K. Effects of fluoride treatment on phosphoric acid-etching in primary teeth: an AFM observation. *Micron* **41**, 498-506, doi:https://doi.org/10.1016/j.micron.2010.02.002 (2010).
37 Chan, Y. L., Ngan, A. H. W. & King, N. M. Nano-scale structure and mechanical properties of the human dentine-enamel junction. *Journal of the Mechanical Behavior of Biomedical Materials* **4**, 785-795, doi:10.1016/j.jmbbm.2010.09.003 (2011).
38 Sun, L., Liang, S., Sa, Y., Wang, Z., Ma, X., Jiang, T. & Wang, Y. Surface alteration of human tooth enamel subjected to acidic and neutral 30% hydrogen peroxide. *Journal of Dentistry* **39**, 686-692, doi:https://doi.org/10.1016/j.jdent.2011.07.011 (2011).
39 Rodríguez-Vilchis, L. E., Contreras-Bulnes, R., Olea-Mejìa, O. F., Sánchez-Flores, I. & Centeno-Pedraza, C. Morphological and structural changes on human dental enamel after Er:YAG laser irradiation: AFM, SEM, and EDS evaluation. *Photomedicine Laser Surgery* **29**, 493-500, doi:10.1089/pho.2010.2925 (2011).
40 Hsu, C. C., Chung, H. Y., Yang, J.-M., Shi, W. & Wu, B. Influence of 8DSS peptide on nano-mechanical behavior of human enamel. *Journal of Dental Research* **90**, 88-92, doi:10.1177/0022034510381904 (2011).
41 Zhao, W., Cao, C. & Korach, C. S. Measurement of structural variations in enamel nanomechanical properties using quantitative atomic force acoustic microscopy. 373-381 (Springer New York, New York, NY, 2011).
42 Cheong, Y., Choi, S., Kim, S. J. & Park, H.-K. Nanostructural effect of acid-etching and fluoride application on human primary and permanent tooth enamels. *Materials Science and Engineering: C* **32**, 1127-1132, doi:https://doi.org/10.1016/j.msec.2012.02.030 (2012).
43 Mahmoud, S. H., Ahmed, M. E., Mahmoud, K. M., Grawish Mel, A. & Zaher, A. R. Effects of phosphoric acid concentration and etching duration on enamel and dentin tissues of uremic patients receiving hemodialysis: an AFM study. *The Journal of Adhesive Dentistry* **14**, 215-221, doi:10.3290/j.jad.a22421 (2012).
44 Sa, Y., Wang, Z., Ma, X., Lei, C., Liang, S., Sun, L., Jiang, T. & Wang, Y. Investigation of three home-applied bleaching agents on enamel structure and mechanical properties: an *in situ* study. *Journal of Biomedical Optics* **17**, 035002 (2012).
45 Cerci, B. B., Roman, L. S., Guariza-Filho, O., Camargo, E. S. & Tanaka, O. M. Dental enamel roughness with different acid etching times: atomic force microscopy study. *European Journal of General Dentistry* **1**, 187-191, doi:10.4103/2278-9626.105385 (2012).

46 Taher, N. M. Atomic force microscopy and tridimensional topography analysis of human enamel after resinous infiltration and storage in water. *Saudi Medical Journal* **34**, 408-414 (2013).
47 Hashimoto, Y., Hashimoto, Y., Nishiura, A. & Matsumoto, N. Atomic force microscopy observation of enamel surfaces treated with selfetching primer. *Dental Materials Journal* **32**, 181-188, doi:10.4012/dmj.2012-227 (2013).
48 Poggio, C., Lombardini, M., Vigorelli, P. & Ceci, M. Analysis of dentin/enamel remineralization by a CPP-ACP paste: AFM and SEM study. *Scanning* **35**, 366-374, doi:10.1002/sca.21077 (2013).
49 Agrawal, N., Shashikiran, N., Singla, S., Ravi, K. & Kulkarni, V. Atomic force microscopic comparison of remineralization with casein-phosphopeptide amorphous calcium phosphate paste, acidulated phosphate fluoride gel and iron supplement in primary and permanent teeth: an *in-vitro* study. *Contemporary Clinical Dentistry* **5**, 75-80, doi:10.4103/0976-237x.128672 (2014).
50 Poggio, C., Ceci, M., Beltrami, R., Lombardini, M. & Colombo, M. Atomic force microscopy study of enamel remineralization. *Annali Di Stomatologia* **5**, 98-102 (2014).
51 Attin, T. & Wegehaupt, F. J. Methods for assessment of dental erosion. in *Erosive Tooth Wear (From Diagnosis to Therapy)* Vol. 25 (ed Ganss C Lussi A) 123-142 (2014), doi:10.1159/000360355.
52 Zhou, C., Zhang, D., Bai, Y. & Li, S. Casein phosphopeptide–amorphous calcium phosphate remineralization of primary teeth early enamel lesions. *Journal of Dentistry* **42**, 21-29, doi:https://doi.org/10.1016/j.jdent.2013.11.005 (2014).
53 Shahmoradi, M., Bertassoni, L. E., Elfallah, H. M. & Swain, M. Fundamental structure and properties of enamel, dentin and cementum. in *Advances in Calcium Phosphate Biomaterials* (ed Besim Ben-Nissan) 511-547 (Springer Berlin Heidelberg, 2014), doi:10.1007/978-3-642-53980-0_17.
54 Lombardini, M., Ceci, M., Colombo, M., Bianchi, S. & Poggio, C. Preventive effect of different toothpastes on enamel erosion: AFM and SEM studies. *Scanning* **36**, 401-410, doi:https://doi.org/10.1002/sca.21132 (2014).
55 Liang, K., Xiao, S., Shi, W., Li, J., Yang, X., Gao, Y., Gou, Y., Hao, L., He, L., Cheng, L., et al. 8DSS-promoted remineralization of demineralized dentin *in vitro*. *Journal of Materials Chemistry B* **3**, 6763-6772, doi:10.1039/C5TB00764J (2015).
56 Liang, K., Yuan, H., Li, J., Yang, J., Zhou, X., He, L., Cheng, L., Gao, Y., Xu, X., Zhou, X., et al. Remineralization of demineralized dentin induced by amine-terminated PAMAM dendrimer. *Macromolecular Materials and Engineering* **300**, 107-117, doi:https://doi.org/10.1002/mame.201400207 (2015).
57 Ulrich, I., Mueller, J., Wolgin, M., Frank, W. & Kielbassa, A. M. Tridimensional surface roughness analysis after resin infiltration of (deproteinized) natural subsurface carious lesions. *Clinical Oral Investigations* **19**, 1473-1483, doi:10.1007/s00784-014-1372-5 (2015).
58 Wang, C., Zhao, Y., Zheng, S., Xue, J., Zhou, J., Tang, Y., Jiang, L. & Li, W. Effect of enamel morphology on nanoscale adhesion forces of streptococcal bacteria : an AFM study. *Scanning* **37**, 313-321, doi:https://doi.org/10.1002/sca.21218 (2015).
59 Xia, J., Zheng, J., Huang, D., Tian, Z. R., Chen, L., Zhou, Z., Ungar, P. S. & Qian, L. New model to explain tooth wear with implications for microwear formation and diet reconstruction. *Proceedings of the National Academy of Sciences* **112**, 10669-10672, doi:10.1073/pnas.1509491112 (2015).
60 Arnold, W. H., Haddad, B., Schaper, K., Hagemann, K., Lippold, C. & Danesh, G. Enamel surface alterations after repeated conditioning with HCl. *Head & Face Medicine* **11**, 32, doi:10.1186/s13005-015-0089-2 (2015).

61 Lucas, P. W. & van Casteren, A. The wear and tear of teeth. *Medical Principles and Practice : International Journal of the Kuwait University, Health Science Centre* **24 Suppl 1**, 3-13, doi:10.1159/000367976 (2015).
62 Abdallah, M.-N., Eimar, H., Bassett, D. C., Schnabel, M., Ciobanu, O., Nelea, V., McKee, M. D., Cerruti, M. & Tamimi, F. Diagenesis-inspired reaction of magnesium ions with surface enamel mineral modifies properties of human teeth. *Acta Biomaterialia* **37**, 174-183, doi:10.1016/j.actbio.2016.04.005 (2016).
63 Arnold, W. H., Meyer, A.-K. & Naumova, E. A. Surface roughness of initial enamel caries lesions in human teeth after resin infiltration. *The Open Dentistry Journal* **10**, 505-515, doi:10.2174/1874210601610010505 (2016).
64 Kilponen, L., Lassila, L., Tolvanen, M., Varrela, J. & Vallittu, P. K. Effect of removal of enamel on rebonding strength of resin composite to enamel. *BioMed Research International* **2016**, 1818939, doi:10.1155/2016/1818939 (2016).
65 Zhao, X., Pan, J., Malmstrom, H. S. & Ren, Y.-F. Protective effects of resin sealant and flowable composite coatings against erosive and abrasive wear of dental hard tissues. *Journal of Dentistry* **49**, 68-74, doi:https://doi.org/10.1016/j.jdent.2016.01.013 (2016).
66 Lin, W. T., Kitasako, Y., Nakashima, S. & Tagami, J. A comparative study of the susceptibility of cut and uncut enamel to erosive demineralization. *Dental Materials Journal* **36**, 48-53, doi:10.4012/dmj.2016-239 (2017).
67 Mirjanić, V. & Mirjanić, Đ. AFM analysis of enamel damage due to etching with orthophosphoric acid. *Contemporary Materials* **1**, 60-72 (2017).
68 Mockdeci, H., Polonini, H., Martins, I., Granato, A.-P., Raposo, N. & Chaves, M.-d. G. Evaluation of *ex vivo* effectiveness of commercial desensitizing dentifrices. *Journal of Clinical and Experimental Dentistry* **9**, e503-e510, doi:10.4317/jced.53040 (2017).
69 Mullan, F., Bartlett, D. & Austin, R. S. Measurement uncertainty associated with chromatic confocal profilometry for 3D surface texture characterization of natural human enamel. *Dental Materials* **33**, e273-e281, doi:https://doi.org/10.1016/j.dental.2017.04.004 (2017).
70 Mullan, F., Austin, R. S., Parkinson, C. R., Hasan, A. & Bartlett, D. W. Measurement of surface roughness changes of unpolished and polished enamel following erosion. *PLoS ONE* **12**, e0182406, doi:10.1371/journal.pone.0182406 (2017).
71 Saeki, K., Chien, Y. C., Nonomura, G., Chin, A. F., Habelitz, S., Gower, L. B., Marshall, S. J. & Marshall, G. W. Recovery after PILP remineralization of dentin lesions created with two cariogenic acids. *Archives of Oral Biology* **82**, 194-202, doi:https://doi.org/10.1016/j.archoralbio.2017.06.006 (2017).
72 Toledano, M., Osorio, E., Aguilera, F. S., Cabello, I., Toledano-Osorio, M. & Osorio, R. *Ex vivo* detection and characterization of remineralized carious dentin, by nanoindentation and single point Raman spectroscopy, after amalgam restoration. *Journal of Raman Spectroscopy* **48**, 384-392, doi:10.1002/jrs.5055 (2017).
73 Yang, X., Yang, B., He, L., Li, R., Liao, Y., Zhang, S., Yang, Y., Xu, X., Zhang, D., Tan, H., et al. Bioinspired peptide-decorated tannic acid for in situ remineralization of tooth enamel: in vitro and in vivo evaluation. *ACS Biomaterials Science & Engineering* **3**, 3553-3562, doi:10.1021/acsbiomaterials.7b00623 (2017).
74 Gyurkovics, M., Baumann, T., Carvalho, T. S., Assunção, C. M. & Lussi, A. *In vitro* evaluation of modified surface microhardness measurement, focus variation 3D microscopy and contact stylus profilometry to assess enamel surface loss after erosive–abrasive challenges. *PLOS ONE* **12**, e0175027, doi:10.1371/journal.pone.0175027 (2017).
75 Meredith, L., Farella, M., Lowrey, S., Cannon, R. D. & Mei, L. Atomic force microscopy analysis of enamel nanotopography after interproximal reduction. *American Journal of Orthodontics and Dentofacial Orthopedics* **151**, 750-757, doi:https://doi.org/10.1016/j.ajodo.2016.09.021 (2017).

76 Mohebi, S. & Ameli, N. Atomic force microscopy analysis of enamel nanotopography after interproximal reduction. *American Journal of Orthodontics and Dentofacial Orthopedics* **152**, 295-296, doi:https://doi.org/10.1016/j.ajodo.2017.06.006 (2017).
77 Xia, J., Tian, Z. R., Hua, L., Chen, L., Zhou, Z., Qian, L. & Ungar, P. S. Enamel crystallite strength and wear: nanoscale responses of teeth to chewing loads. *Journal of the Royal Society Interface* **14**, 1-8, doi:10.1098/rsif.2017.0456 (2017).
78 Baysan, A., Sleibi, A., Ozel, B. & Anderson, P. The quantification of surface roughness on root caries using Noncontact Optical Profilometry—An in vitro study. *Lasers in Dental Science* **2**, 229-237, doi:10.1007/s41547-018-0041-4 (2018).
79 Mullan, F., Mylonas, P., Parkinson, C., Bartlett, D. & Austin, R. S. Precision of 655 nm Confocal Laser Profilometry for 3D surface texture characterisation of natural human enamel undergoing dietary acid mediated erosive wear. *Dental Materials* **34**, 531-537, doi:https://doi.org/10.1016/j.dental.2017.12.012 (2018).
80 Feroz, S., Aamir, S. & Nawabi, S. Susceptibility of human deciduous enamel to erosive wear after exposure to commonly prescribed oral pediatric liquid medicaments: an AFM based *in vitro* analysis. *International Journal of Dental Sciences and Research* **6**, 138-142 (2018).
81 Ijbara, M., Wada, K., Tabata, M. J., Wada, J., Go Inoue, G. & Miyashin, M. Enamel microcracks induced by simulated occlusal wear in mature, immature, and deciduous teeth. *BioMed Research International* **2018**, 1-9 (2018).
82 Liu, Y., Ding, C., He, L., Yang, X., Gou, Y., Xu, X., Liu, Y., Zhao, C., Li, J. & Li, J. Bioinspired heptapeptides as functionalized mineralization inducers with enhanced hydroxyapatite affinity. *Journal of Materials Chemistry B* **6**, 1984-1994, doi:10.1039/C7TB03067C (2018).
83 Mohamed, A. M., Wong, K. H., Lee, W. J., Marizan Nor, M., Mohd Hussaini, H. & Rosli, T. I. *In vitro* study of white spot lesion: maxilla and mandibular teeth. *The Saudi Dental Journal* **30**, 142-150, doi:https://doi.org/10.1016/j.sdentj.2017.12.001 (2018).
84 Sui, T., Dluhoš, J., Li, T., Zeng, K., Cernescu, A., Landini, G. & Korsunsky, A. M. Structure-function correlative microscopy of peritubular and intertubular dentine. *Materials* **11**, doi:10.3390/ma11091493 (2018).
85 Sorozini, M., dos Reis Perez, C. & Rocha, G. M. Enamel sample preparation for AFM: influence on roughness and morphology. *Microscopy Research and Technique* **81**, 1071-1076, doi:10.1002/jemt.23073 (2018).
86 Shao, C., Jin, B., Mu, Z., Lu, H., Zhao, Y., Wu, Z., Yan, L., Zhang, Z., Zhou, Y., Pan, H., et al. Repair of tooth enamel by a biomimetic mineralization frontier ensuring epitaxial growth. *Science Advances* **5**, eaaw9569, doi:10.1126/sciadv.aaw9569 (2019).
87 Li, P., Oh, C., Kim, H., Chen-Glasser, M., Gun, P., Jetybayeva, A., Yeom, J., Kim, H., Ryu, J. & Hong, S. Nanoscale characterization of the impact of beverages on the enamel surface of human teeth. *arXiv preprint arXiv:1909.02419*, 1-17 (2019).
88 Liang, K., Wang, S., Tao, S., Xiao, S., Zhou, H., Wang, P., Cheng, L., Zhou, X., Weir, M. D., Oates, T. W., et al. Dental remineralization via poly(amido amine) and restorative materials containing calcium phosphate nanoparticles. *International Journal of Oral Science* **11**, 15, doi:10.1038/s41368-019-0048-z (2019).
89 Sereda, G., VanLaecken, A. & Turner, J. A. Monitoring demineralization and remineralization of human dentin by characterization of its structure with resonance-enhanced AFM-IR chemical mapping, nanoindentation, and SEM. *Dental Materials* **35**, 617-626, doi:https://doi.org/10.1016/j.dental.2019.02.007 (2019).
90 Shimomura, N., Tanaka, R., Shibata, Y., Zhang, Z., Li, Q., Zhou, J., Wurihan, Tobe, T., Ikeda, S., Yoshikawa, K., et al. Exceptional contact elasticity of human enamel in nanoindentation test. *Dental Materials* **35**, 87-97, doi:https://doi.org/10.1016/j.dental.2018.11.005 (2019).
91 Solaymani, S., Ţălu, Ş., Ghoranneviss, M., Elahi, S. M., Shafiekhani, A., Hantehzadeh, M. & Nezafat, N. B. Multifractal analysis of human canine teeth at nano scale: atomic force

microscopy studies. *International Nano Letters*, 1-8, doi:10.1007/s40089-019-00293-7 (2019).
92 Arango-Santander, S., Montoya, C., Pelaez-Vargas, A. & Ossa, E. A. Chemical, structural and mechanical characterization of bovine enamel. *Archives of Oral Biology* **109**, 104573, doi:https://doi.org/10.1016/j.archoralbio.2019.104573 (2020).
93 Lei, L., Zheng, L., Xiao, H., Zheng, J. & Zhou, Z. Wear mechanism of human tooth enamel: the role of interfacial protein bonding between HA crystals. *Journal of the Mechanical Behavior of Biomedical Materials* **110**, 103845, doi:https://doi.org/10.1016/j.jmbbm.2020.103845 (2020).
94 Danesh, G., Podstawa, P. K. K., Schwartz, C.-E., Kirschneck, C., Bizhang, M. & Arnold, W. H. Depth of acid penetration and enamel surface roughness associated with different methods of interproximal enamel reduction. *PLoS ONE* **15**, e0229595, doi:10.1371/journal.pone.0229595 (2020).
95 Tsenova-Ilieva, I. & Karova, E. Application of atomic force microscopy in dental investigations. *International Journal of Science and Research (IJSR)* **9**, 1319-1326, doi:10.21275/SR20323220034 (2020).
96 Huang, L., Zhang, X., Shao, J., Zhou, Z., Chen, Y. & Hu, X. Nanoscale chemical and mechanical heterogeneity of human dentin characterized by AFM-IR and bimodal AFM. *Journal of Advanced Research* **22**, 163-171, doi:https://doi.org/10.1016/j.jare.2019.12.004 (2020).
97 Sadyrin, E., Swain, M., Mitrin, B., Rzhepakovsky, I., Nikolaev, A., Irkha, V., Yogina, D., Lyanguzov, N., Maksyukov, S. & Aizikovich, S. Characterization of enamel and dentine about a white spot lesion: mechanical properties, mineral density, microstructure and molecular composition. *Nanomaterials* **10**, 1889 (2020).
98 Sugsompian, K., Tansalarak, R. & Piyapattamin, T. Comparison of the enamel surface roughness from different polishing methods: scanning electron microscopy and atomic force microscopy investigation. *European journal of dentistry* **14**, 299-305, doi:10.1055/s-0040-1709945 (2020).
99 Świetlicka, I., Kuc, D., Świetlicki, M., Arczewska, M., Muszyński, S., Tomaszewska, E., Prószyński, A., Gołacki, K., Błaszczak, J., Cieślak, K., et al. Near-surface studies of the changes to the structure and mechanical properties of human enamel under the action of fluoride varnish containing CPP–ACP compound. *Biomolecules* **10**, 765 (2020).
100 Cherian, T., Subramaniam, P. & Gupta, M. Erosive effect of milk, honey, cereal porridge, and millet porridge on enamel of primary teeth: an *in vitro* study. *Indian Journal of Dental Research* **31**, 129-133, doi:10.4103/ijdr.IJDR_224_18 (2020).
101 Wu, Q., Mei, M. L., Wu, X., Shi, S., Xu, Y., Chu, C. H. & Chen, Y. Remineralising effect of 45S5 bioactive glass on artificial caries in dentine. *BMC oral health* **20**, 49-49, doi:10.1186/s12903-020-1038-4 (2020).
102 McGrath, K., Limmer, L. S., Lockey, A.-L., Guatelli-Steinberg, D., Reid, D. J., Witzel, C., Bocaege, E., McFarlin, S. C. & El Zaatari, S. 3D enamel profilometry reveals faster growth but similar stress severity in Neanderthal versus *Homo sapiens* teeth. *Scientific Reports* **11**, 522, doi:10.1038/s41598-020-80148-w (2021).
103 Solaymani, S., Nezafat, N. B., Ţălu, Ş., Shafiekhani, A., Dalouji, V., Amiri, A., Rezaee, S. & Morozov, I. A. Atomic force microscopy studies of enamel, inner enamel, dentin, and cementum in canine teeth. *Microscopy Research and Technique*, 1-8, doi:https://doi.org/10.1002/jemt.23668 (2021).
104 Yu, F., Luo, M. L., Xu, R. C., Huang, L., Yu, H. H., Meng, M., Jia, J. Q., Hu, Z. H., Wu, W. Z., Tay, F. R., et al. A novel dentin bonding scheme based on extrafibrillar demineralization combined with covalent adhesion using a dry-bonding technique. *Bioactive Materials* **6**, 3557-3567, doi:https://doi.org/10.1016/j.bioactmat.2021.03.024 (2021).
105 Gheorghe, G. F., Amza, O. E., Dimitriu, B., Garneata, L., Suciu, I., Moldovan, M., Chisnoiu, R. M., Prodan, D. & Chisnoiu, A. M. Comparative in vitro studies on the effect of bleaching

agents on dental structures in healthy and predialysis patients. *Applied Sciences* **11**, 7807 (2021).

106 Machoy, M., Wilczyński, S., Szyszka-Sommerfeld, L., Woźniak, K., Deda, A. & Kulesza, S. Mapping of nanomechanical properties of enamel surfaces due to orthodontic treatment by AFM method. *Applied Sciences* **11**, 3918 (2021).

107 Naeem, Z. J., Salman, A. M., Faris, R. A. & Al-Janabi, A. Highly efficient optical fiber sensor for instantaneous measurement of elevated temperature in dental hard tissues irradiated with an Nd:YaG laser. *Applied Optics* **60**, 6189-6198, doi:10.1364/AO.431369 (2021).

108 Mao, J., Wang, L., Jiang, Y., Cheng, H., Li, N., Shi, S., Fan, F., Ma, J. & Huang, S. Nanoscopic wear behavior of dentinogenesis imperfecta type II tooth dentin. *Journal of the Mechanical Behavior of Biomedical Materials* **120**, 104585, doi:https://doi.org/10.1016/j.jmbbm.2021.104585 (2021).
